# Microplastic Effect Tests Should Use a Standard Heterogeneous Mixture: Multifarious Impacts among 16 Benthic Invertebrate Species Detected under Ecologically Relevant Test Conditions

**DOI:** 10.1021/acs.est.3c06829

**Published:** 2023-11-22

**Authors:** Vera N. de Ruijter, Matthias Hof, Petranta Kotorou, Jesse van Leeuwen, Martine J. van den Heuvel-Greve, Ivo Roessink, Albert A. Koelmans

**Affiliations:** † Aquatic Ecology and Water Quality Management Group, Wageningen University, Post Office Box 47, 6700 AA Wageningen, Netherlands; ‡ Wageningen Marine Research, Wageningen University & Research, Post Office Box 77, 4400 AB Yerseke, Netherlands; § Wageningen Environmental Research, Wageningen University & Research, Post Office Box 47, 6700 AA Wageningen, Netherlands

**Keywords:** risk assessment, reference material, standard test, benthic species, freshwater, marine

## Abstract

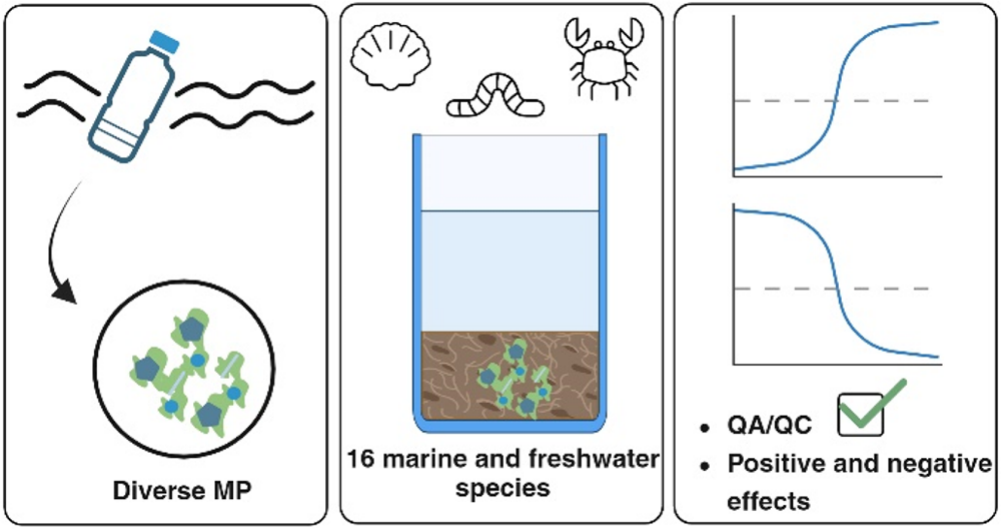

Microplastics require a risk assessment framework that takes their multidimensionality into account while exclusively considering robust data. Therefore, effect tests should use a diverse, environmentally relevant microplastic (ERMP) standard material that adheres to high-quality requirements. In this study, we provide chronic dose–effect relationships and effect thresholds for 16 benthic species exposed to ERMP. The ERMP was created from plastic items collected from natural sources and cryogenically milled to represent the diversity of microplastics. The test design met 20 previously published quality assurance and quality control criteria. Adverse effect thresholds (EC_10_) were determined at ERMP concentrations of 0.11 ± 0.17% sediment dry weight (*Gammarus pulex*, growth), 0.49 ± 0.68% sediment dry weight (*Lumbriculus variegatus*, growth), and 1.90 ± 1.08% sediment dry weight (*L. variegatus*, reproduction). A positive effect of microplastics, such as decreased mortality, was observed for *Cerastoderma edule* (EC_10_ = 0.021 ± 0.027% sediment dry weight) and *Sphaerium corneum* (EC_10_ = 7.67 ± 3.41% sediment dry weight), respectively. Several of these laboratory-based single-species effect thresholds for ERMP occurred at concentrations lower than those found in the environment. For other species, no significant effects were detected up to an ERMP dose of 10% dry weight.

## Abstract 2

Effects of microplastics are assessed for a range of 16 benthic invertebrate species using strict quality criteria and an environmentally relevant standard microplastic test material.

## Introduction

In recent years, research into microplastics has increased significantly as a result of growing concerns within the public, political, and scientific communities. It is well-known now that microplastics can be found anywhere and could potentially lead to negative impacts on aquatic ecosystems.^1−3^ Although sediments are considered sinks for microplastics, most research to date has focused on pelagic exposure scenarios. Moreover, not much is known about differences between habitats (marine versus freshwater) or about the implications of species traits on the toxicity of microplastics. Prospective risk assessments show that it is only a matter of time until the risk of adverse effects on the ecosystem function will increase and that this is already the case for some hotspot sites, such as the Mediterranean, the Yellow Sea, and several highly polluted freshwater sites.^4−7^ The results of these risk assessments should however be regarded as preliminary because they were not performed in a regulatory context and the ecotoxicological data used are surrounded with uncertainties.^1,6,8^ Many researchers acknowledge that methodologies in plastic research need improvement and harmonization.^1,4,9−13^ Moreover, there is an urgent need to deal with the complexity of microplastics as a diverse contaminant, varying in size, shape, and polymer type, and to elucidate the underlying mechanisms of effects to explain conflicting results.^14,15^ Recently developed species sensitivity distributions (SSDs) contain ecotoxicological data that do not test microplastics in their diversity as one would encounter in the aquatic environment.^4,5,8,16^ Because data points in these SSDs relate to different particle types, results from such SSDs are fundamentally flawed. Rescaling methods have been developed that solve the problem of this misalignment,^6,17^ and these have been applied to derive management thresholds for the first time in a regulatory context.^18,19^ While elegant and efficient and considered the best option at the moment, they use several approaches and assumptions that introduce new uncertainties.^6^ In addition, they often have to use relatively poor-quality input data,^19^ a problem that cannot be completely solved by scaling or alignment methods. We propose that, to increase the realism of risk assessments, exposure and effect threshold data should be used that meet the highest quality assurance and quality control (QA/QC) standards and that relate to environmentally relevant standard microplastic mixtures that reflect the full multidimensionality of the material.^3,17^ More specifically, for effect tests, microplastic mixtures used should provide a valid representation of the distribution of polymer types, shapes, and sizes as found in nature,^1^ so that as little extrapolation via rescaling as possible is needed.

This study aimed to provide threshold effect concentrations for a range of benthic species with different feeding traits and habitats for risk assessment purposes. To achieve this aim, we first prepared an environmentally relevant microplastic (ERMP) mixture and then tested 16 invertebrate species using that mixture while fulfilling strict QA/QC criteria.^1^ For instance, key features of the quality assurance were that ERMP was characterized extensively, contamination was minimized, exposure concentrations were homogeneous and verified, natural particles were included, biofouling of microplastics was allowed to increase environmental relevance,^20,21^ environmentally relevant concentrations were used, and six replicated doses were used to enable dose–response modeling to detect and report effect thresholds. Effect data were analyzed using dose–response models, and generalized linear mix models were applied to explore differences in effects between marine versus freshwater species and between feeding traits.

## Materials and Methods

### Quality Assurance and Quality Control (QA/QC)

Test design, materials, handling of materials, control of background contamination, and exposure conditions fulfilled the 20 QA/QC criteria as defined by de Ruijter et al. A summary of how these criteria were met is provided in Table S1 of the Supporting Information. Additionally, a detailed description of how the criteria verification of the exposure concentration and background contamination were met is provided on pages S2 and S3 of the Materials and Methods of the Supporting Information.

### Preparation of Environmentally Relevant Microplastics

Microplastic particles with varying polymer types, sizes, shapes, and colors were created in the laboratory. In brief, naturally aged macroplastics were collected from the river banks of the National Park Biesbosch (Netherlands), analyzed with attenuated total reflectance Fourier transform infrared spectroscopy (ATR–FTIR) to determine the polymer identity, and subsequently cryogenically milled (Figures S1–S4 of the Supporting Information). Sieve fractions of the milling product were combined in such a way that the final mixture demonstrably resembles ERMP occurring in sediments (Figure S5 of the Supporting Information).^1,22^ The result was an ERMP standard test mixture, with polymer weight percentages of irregular polyethylene (PE) fragments (34%), irregular polypropylene (PP) fragments (15.9%), PP fibers (10.5%), irregular polyethylene terephthalate (PET) fragments (20.6%), and irregular polystyrene (PS) fragments (19%). These proportions are similar to those found in the environment.^22^ Moreover, the ERMP test mixture had a size range between 9 and 5386 μm and a power law slope α (α = 3.28 ± 0.02) (Figure S5) equal to the slope found for microplastic mixtures in freshwater sediments (α = 3.25 ± 0.19).^17^ A detailed description of the preparation and characterization of the ERMP test mixture is provided on pages S4 and S5 of the Materials and Methods and Figures S5 and S6 of the Supporting Information.

### Test Organisms

A total of 16 benthic invertebrate species, comprising 9 freshwater and 7 marine species, were tested in this study. Freshwater species selected were *Gammarus pulex*, *Hyalella azteca*, *Asellus aquaticus*, *Sphaerium corneum*, *Corbicula fluminalis*, *Potamopyrgus antipodarum*, *Tubifex* spp., *Lumbriculus variegatus*, and *Chironomus riparius*. Organisms were selected to differ in their feeding and living behavior; however, they are all connected to the benthic environment (Table S2 of the Supporting Information). Marine species selected were *Alitta virens*, *Limecola balthica*, *Corophium volutator*, *Arenicola marina*, *Cerastoderma edule*, *Porcellana platycheles*, and *Mytilus edulis* (Table S3). A detailed description of the origin and collection of the test organisms is provided on page S6 of the Materials and Methods of the Supporting Information.

### Sediment

Clean freshwater sediments were collected from the experimental field station of Wageningen University (Sinderhoeve, Renkum, Netherlands). Marine sediments were collected from Oesterput and Roelshoek (Eastern Scheldt, Netherlands) and mixed with a ratio of 1:4.^23^ For a detailed description of the collection and preparation of the sediments, see page S7 of the Materials and Methods of the Supporting Information.

### Experimental Setup

The systematic testing approach in this study is similar to that followed by Redondo-Hasselerharm et al., with a few adjustments.^23^ Sediment–microplastic mixtures were added to sediment at concentrations of 0, 0.1, 0.3, 1.0, 2.5, 5.0, and 10.0% dry weight (dw). This translated to a particle concentrations of 5.9 × 10^6^, 1.8 × 10^7^, 5.9 × 10^7^, 1.5 × 10^8^, 2.9 × 10^8^, and 5.9 × 10^8^/kg of dw sediment, respectively. In total, 17 chronic, single-species bioassays were performed. The systematic testing approach in this study is similar to that followed by Redondo-Hasselerharm et al.; however, in the previous study, the sediment that originated from a non-contaminated ditch in Veenkampen (Wageningen, Netherlands) had a total organic matter (TOM) content of 31.6 ± 3.5% (*n* = 4).^23^ While environmentally relevant, this high TOM content could mask the adverse effects of microplastics. After all, negative effects observed in aquatic organisms have been explained by the inhibition of food assimilation and/or decreased nutritional value of food, more commonly referred to as “food dilution”.^1^ Hence, we chose sediment with a lower, more common TOM content. To verify comparability between our testing approaches and implement a positive control, we repeated the previous experiment by Redondo-Hasselerharm et al. with *G. pulex*: once with the lower TOM content sediment and PS fragments as used by Redondo-Hasselerharm et al. and once with the lower TOM content and with ERMP instead of PS fragments. For experiments 1 and 2, experimental units were made by either adding PS fragments or ERMP without PS fragments to sediment (Tables S2 and S3 of the Supporting Information) in the following concentrations 0, 0.5, 1, 3, 5, 10, and 20 wt %. For experiments 3–17, ERMP including PS fragments (Tables S2 and S3 of the Supporting Information) was added to the sediment in the following concentrations 0, 0.1, 0.3, 1.0, 2.5, 5.0, and 10.0 wt %. Concentrations ranging from environmentally relevant (0–1.0%)^24−26^ to high concentrations (2.5–20%) were included to cover criteria related to relevance as well as statistical rigor in finding an effect threshold.^1^

Sediment–microplastic mixtures were manually homogenized with a stainless-steel spoon. Consequently, Dutch standard water (DSW) or filtered seawater was gently added at a 3:1 water/sediment ratio. Experimental units were made in quadruplicate, and four blanks (containing only DSW or seawater) were added to measure background microplastic contamination, e.g., from air fallout. Systems were randomized and left to acclimatize to allow for biofilm formation for 2 weeks before adding the organisms.^20,27−30^ Experimental units received 11–22 organisms depending upon the size of the organisms (Tables S2 and S3 of the Supporting Information). Exposure lasted 28 days. Dissolved oxygen, pH, temperature, conductivity/salinity, and NH_3_ concentrations were measured twice a week, and DSW and seawater were refreshed periodically (Tables S4 and S5 of the Supporting Information). For a detailed description of the experimental design, the reader is referred to page S8 of the Materials and Methods of the Supporting Information.

### End Point Mortality, Reproduction, Growth, Feeding Rate, Emergence, and Development Rate

After 28 days, organisms were sieved, counted, and transferred to clean DSW or seawater to clear their gut for 24 h. Consequently, organisms were rinsed with Milli-Q water and microplastic particles in their gut contents, and body tissues were stored separately at −20 °C for later microplastic analysis. Findings related to microplastic ingestion will be detailed in a companion paper. Organisms were photographed, and either their length was measured using ImageJ^31^ or they were weighed per replica. The emergence of *C. riparius* was counted daily. The feeding rate (mg of dw of leaf organism^–1^ day^–1^) of *G. pulex* was calculated from the loss of poplar leaves (equation S1 of the Supporting Information). An overview of end points measured per organism is provided in Tables S2 and S3 of the Supporting Information.

### Data Analysis

Selection of the best fitting dose–response models and detection of statistically significant EC_50_ threshold effect concentrations were done using the dose–response curve (DRC) package in R.^32^ The significance of ERMP dose dependence (*p*_noEffect_) was assessed using a log-likelihood ratio test using the best fit dose–response model compared to a linear regression model with a slope of 0, representing the absence of dose dependency.

To explore whether the effects of the ERMP concentration on mortality were different for the freshwater versus marine species tested, a generalized linear mixed model (GLMM) was used. Similarly, a GLMM was used to explore whether the effects of ERMP on mortality were different for the feeding traits (filter feeders, sediment/deposit feeders, sediment grazers, and facultative deposit feeders). All statistical analyses and graphs were performed in RStudio.^33^ For a detailed description of the statistical approach, the reader is referred to pages S9 and S10 of the Materials and Methods of the Supporting Information.

## Results and Discussion

### Mortality, Emergence, and Reproduction

The mortality in the controls was 20.0% on average across all tests, ranging from 0.0% (*P. platycheles* and *C. fluminalis*) to 75.0% (*C. edule*), with a median mortality of 9.5% (Tables S2 and S3 of the Supporting Information). The mean total number of *C. riparius* that did not emerge was 40%. Additionally, *L. variegatus* had a mean reproduction factor of 2.9, which is more than acceptable.^34^ Some marine species were never tested before, making control mortality less predictable and, in some cases, less ideal in the context of effect testing for regulatory purposes. Error in replicated treatments was fairly high in some cases. We observed a cascading phenomenon in the test systems of *C. edule*, *P. platycheles*, *A. marina*, and *M. edulis*, implying that the death and decay of only one individual resulted in a decline in water quality, which, in turn, resulted in the death of more individuals. Note that the effect thresholds derived from effect tests with high mortalities in the controls are not fit for regulatory purposes. Nevertheless, sediments were non-toxic, water quality parameters were good (Tables S5 and S6 of the Supporting Information), and 80% of all individuals tested survived 28 days of exposure, illustrating that test conditions were generally sufficient.

**Figure 1:**
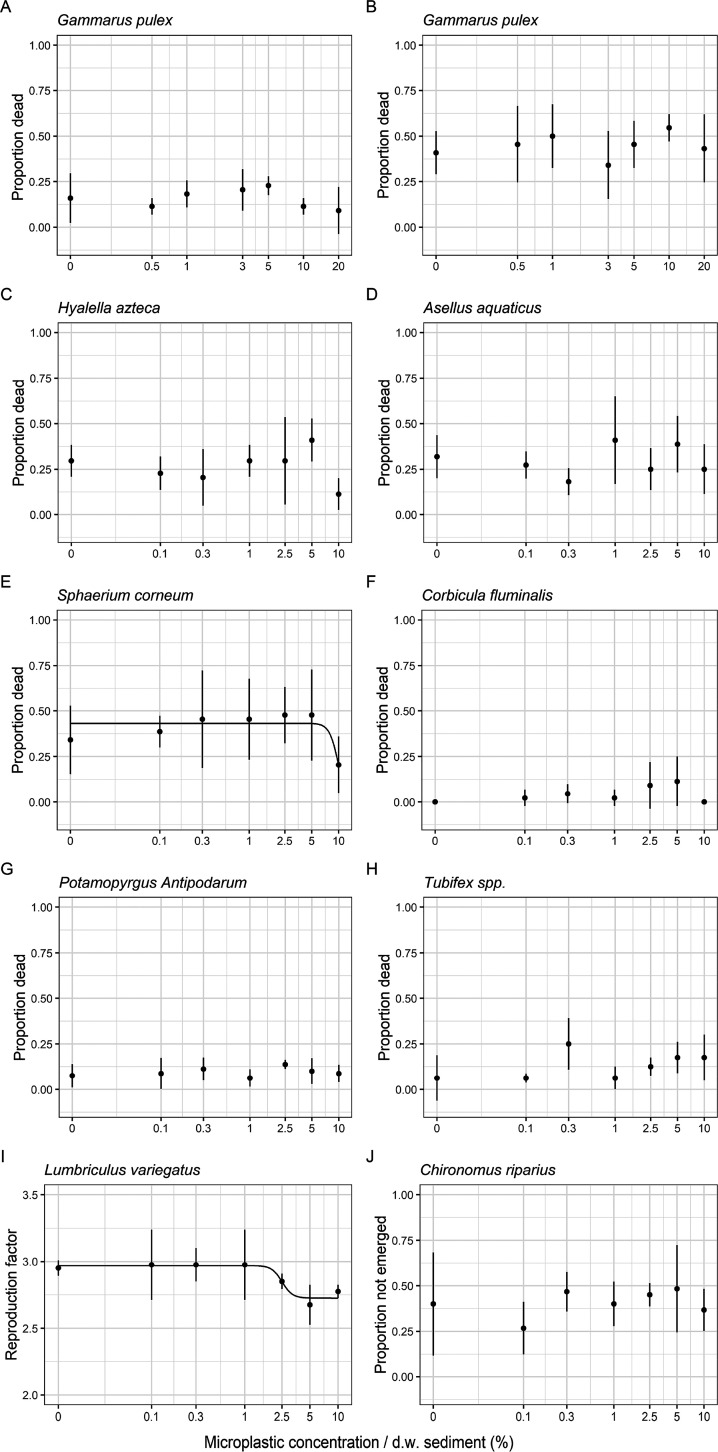
Mean mortality [±standard deviation (sd)] expressed in proportion (dead/start population) of (A) *G. pulex* exposed to PS and (B) *G. pulex* exposed to ERMP with concentrations up to 20% in dw sediment, (C) *H. azteca*, (D) *A. aquaticus*, (E) *S. corneum*, (F) *C. fluminalis*, (G) *P. antipodarum*, and (H) *Tubifex* spp. exposed to ERMP + PS with concentrations up to 10% in dw sediment, (I) mean reproduction factor (±sd) of *L. variegatus* exposed to ERMP + PS, and (J) mean total emergence (±sd) of *C. riparius* exposed to ERMP + PS. The exposure time for all freshwater species was 28 days. Three- and four-parameter log–logistic dose–response models were plotted for (E) *S. corneum* and (I) *L. variegatus*, respectively. Concentrations are on a log scale. The zero concentration has been converted to 0.01 to allow for plotting on the log scale.

**Figure 2:**
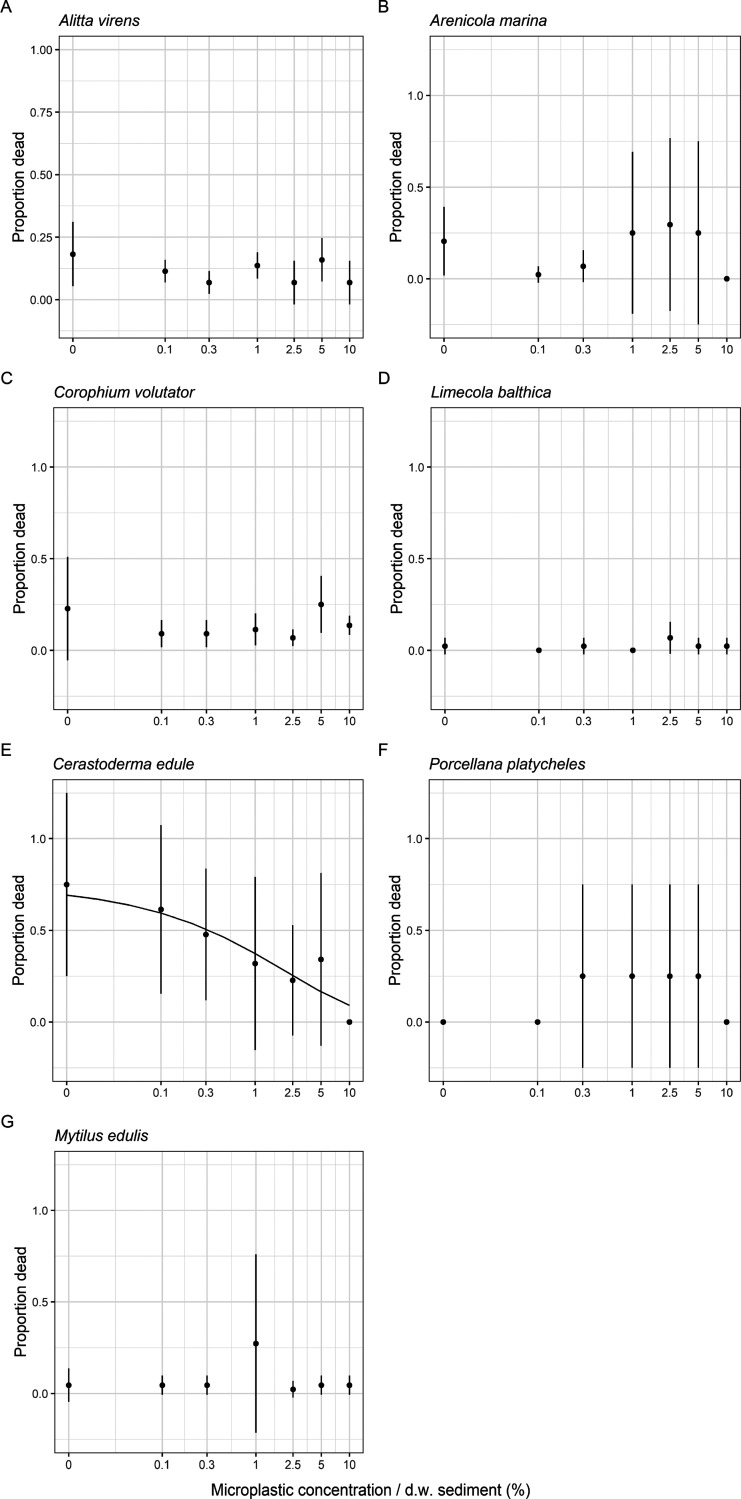
Mean mortality (±sd) expressed in proportion (dead/start population) of (A) *A. virens*, (B) *A. marina*, (C) *C. volutator*, (D) *L. balthica*, (E) *C. edule*, (F) *P. platycheles*, and (G) *M. edulis* exposed to ERMP + PS with concentrations up to 20% in dw sediment. The exposure time for all marine species was 28 days. A three-parameter Weibull (type 1) model was plotted for (E) *C. edule*. Note that concentrations are on a log scale. Additionally, the zero concentration has been converted to 0.01 to allow for plotting on the log scale.

There was no statistically significant effect threshold found for mortality of *G. pulex* after chronic exposure to ERMP without PS fragments with concentrations up to 20% dw (Figure 1 and Table S7 of the Supporting Information). There were no statistically significant effect thresholds found for the marine and freshwater species *H. azteca*, *A. aquaticus*, *C. fluminalis*, *P. antipodarum*, *Tubifex* spp., *A. virens*, *L. balthica*, *C. volutator*, *A. marina*, *P. platycheles*, or *M. edulis* after chronic exposure to ERMP with concentrations up to 10% dw in sediment (Figures 1 and 2 and Tables S7 and S8 of the Supporting Information). Statistically significant effect thresholds were found for mortality for the species *G. pulex* after chronic exposure to PS; however, the dose–response curve was not significant (Figure 1 and Table S7 of the Supporting Information). Moreover, a significant effect threshold was found after chronic exposure to ERMP with concentrations up to 10% dw in sediment for the emergence of *C. riparius*; however, the dose–response curve was not significant (Figure 1 and Table S7 of the Supporting Information). A significant dose–response curve, however, was found for the reproduction of *L. variegatus* (*p* = 0.009; Table S7 of the Supporting Information and Figure 1). Dose–response curve fitting yielded a significant EC_50_ value of 2.51 ± 0.44% (*p* = 0.002) for *L. variegatus* (Figure 1, Table S7 of the Supporting Information, and Table 1).

**Table 1:** Overview of Detected EC_50_ and EC_10_ [±Standard Error (SE)] Threshold Effect Concentrations^a^

organism	aquatic environment	end point	adverse effect	type of DRC^a^ fitted	EC_10_ ± SE (%)	EC_10_ microplastics/kg of dw sediment	EC_50_ ± SE (%)	EC_50_ microplastics/kg of dw sediment	*p* value effect threshold^b^	*p* value log-likelihood ratio test
*Gammarus pulex* (ERMP–PS)^c^	freshwater	growth	yes	LL.2	0.11 ± 0.17	6.48 × 10^6^	2.85 ± 1.39	1.68 × 10^8^	0.050*	6.28 × 10^–4^***
*Sphaerium corneum*	freshwater	mortality	no	LL.3	7.67 ± 3.41	4.52 × 10^8^	9.88 ± 0.68	5.82 × 10^8^	2.0 × 10^–16^***	0.013*
*Lumbriculus variegatus*	freshwater	reproduction factor	yes	LL.4	1.90 ± 1.08	1.12 × 10^8^	2.51 ± 0.44	1.48 × 10^8^	0.002**	0.009**
*Lumbriculus variegatus*	freshwater	growth	yes	W1.4	0.49 ± 0.68	2.88 × 10^7^	0.77 ± 0.14	4.53 × 10^7^	0.007**	2.32 × 10^–03^**
*Cerastoderma edule*	marine	mortality	no	W1.3	0.021 ± 0.027	1.24 × 10^6^	1.01 ± 0.45	5.95 × 10^7^	0.003**	3.22 × 10^–15^***

a The dose–response curve package in R provides the following models: Weibull type I model (W1.*x*) and log logistic (LL.*x*), with *x* giving the number of parameters fitted.

b Significant findings (*p* < 0.05) are highlighted in bold. Significance codes: “∗∗∗”, <0.001; “∗∗”, <0.01; and “∗”, <0.05.

c For *G. pulex* exposed to ERMP–PS, a high mortality of 40.9% was found in the control. The effect thresholds for this species is, therefore, fit for regulatory purposes.

We emphasize that it is challenging to compare these results to previous literature data because, unlike the present study, previous data were obtained with microplastics that are not environmentally relevant and previous effect tests often failed to meet sufficient QA/QC criteria. In this sense, the current data can be considered the first of its kind and can only be compared to previous test results with caution.

Nevertheless, our results do not completely contradict previous microplastic experiments with benthic macroinvertebrates in terms of mortality.^23,35−37^ For instance, Imhof and Laforsch tested an environmentally relevant mixture of irregular PA, PET, PC, PS, and polyvinyl chloride (PVC) particles ranging from 4.64 to 602 μm and did not find any effects on morphological or life history parameters of the mud snail *Potamopyrgus antipodarum*.^36^ Additionally, Redondo-Hasselerharm et al. tested a wide size range of irregular PS fragments on *G. pulex*, *H. azteca*, *A. aquaticus*, and *Tubifex* spp. with a dose up to 40% in sediment dw and reported no effects on mortality. Interestingly, they also reported no effects on the reproduction factor of *L. variegatus*, whereas we detected effects in the present study. Several factors may explain the difference. First of all, our present study used a more realistic microplastic mixture with a more diverse polymer composition, whereas shapes were also more diverse, e.g., including fibers and irregularly shaped PE, PP, and PET fragments ranging from 9 to 5386 μm. The inclusion of fibers associated with a longer gut retention time in the current study may increase the impact of microplastics, as also seen in other studies.^38,39^ Second, our present study used sediment with a lower organic matter content (6.8 ± 0.42%) compared to the sediment used by Redondo-Hasselerharm et al. (31.6 ± 3.5%). This suggests that the inclusion of naturally occurring organic particles can lower the impact of microplastics significantly or even mask adverse effects, as also observed in other studies.^20,21,40,41^

However, these explanations of observed differences in adverse effects remain speculative, and the ingestion of ERMP by the organisms as well as effects at different food quality levels need further study to be conclusive. Nevertheless, the present study underlines the importance of testing a set of diverse microplastics to make accurate predictions at the population level. For instance, Silva et al. did not find any adverse effects on the reproduction of *L. variegatus* after long-term exposure to solely irregular-shaped PE, suggesting no negative impacts on *L. variegatus* population fitness.^42^ However, using a diverse suite of environmentally relevant microplastics, we show that the organism *L. variegatus* is likely to be affected at the population level (EC_10_ = 1.90 ± 1.08%; *p* = 8.1 × 10^–6^; Table 1).

Remarkably, a significant positive effect on survival was found for the marine clam *C. edule* (EC_50_ = 1.01 ± 0.45%; *p* = 0.003) and the freshwater clam *S. corneum* (EC_50_ = 9.88 ± 0.68%; *p* = 2.0 × 10^–16^; Tables S7 and S8 of the Supporting Information and Table 1). Note that mortality in the controls was high for *C. edule* (75%), meaning that the control, unlike those for the other species, was not representative of a habitat with optimal conditions. It does however indicate that, with an increasing concentration of ERMP in the sediment, the mortality decreased and a habitat quality for these species increased with the concentration given in the test conditions. *C. edule* and *S. corneum* inhabit the surface of sediments, burrowing to a depth up to 8 cm.^43^ A possible explanation is that the sediment was too compact for these species, causing resistance for burrowing in the top sediment layer. The microplastic amendments may have loosened sediment consolidation, reducing the energy required for digging. It remains unclear to what extent this effect would be detectable in nature, where sediment top layers are more dynamic as a result of bioturbation and wind- or wave-induced pressure gradients. Moreover, on an intertidal flat in nature, *C. edule* would be able to move to a location with more optimal sediment.

### Growth and Feeding Activity

**Figure 3:**
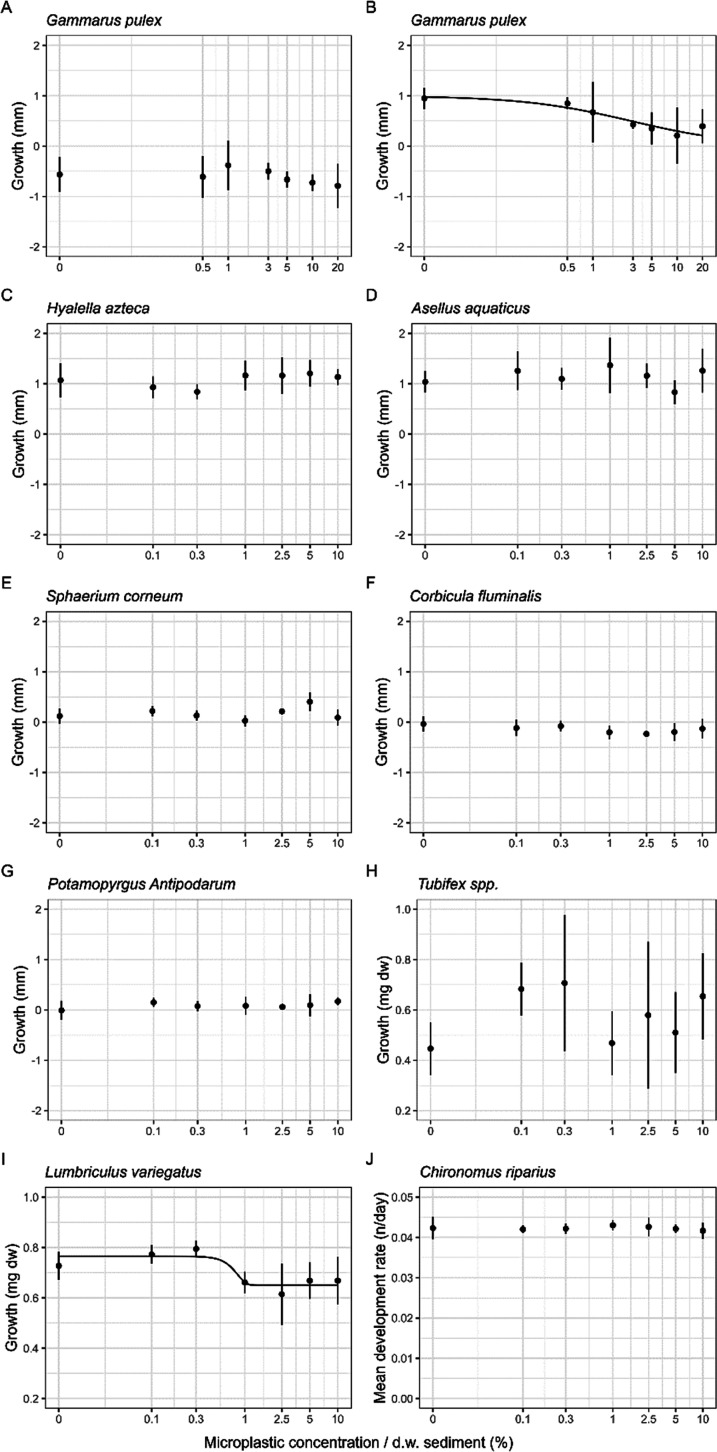
Mean growth (±sd) expressed in length (mm) of (A) *G. pulex* exposed to PS and (B) *G. pulex* exposed to ERMP with concentrations up to 20% in dw sediment, mean growth (±sd) expressed in millimeters or milligrams of (C) *H. azteca*, (D) *A. aquaticus*, (E) *S. corneum*, (F) *C. fluminalis*, (G) *P. antipodarum*, (H) *Tubifex* spp., and (I) *L. variegatus* exposed to ERMP + PS with concentrations up to 10% in dw sediment, and mean development rate (±sd) of (J) *C. riparius* exposed to ERMP + PS with concentrations up to 10% in dw sediment. The exposure time for all freshwater species was 28 days. A two-parameter log–logistic dose–response model was plotted for (B) *G. pulex* exposed to ERMP–PS, respectively. A four-parameter Weibull (type 1) model was plotted for (I) *L. variegatus*. Note that concentrations are on a log scale. Additionally, the zero concentration has been converted to 0.01 to allow for plotting on the log scale.

**Figure 4:**
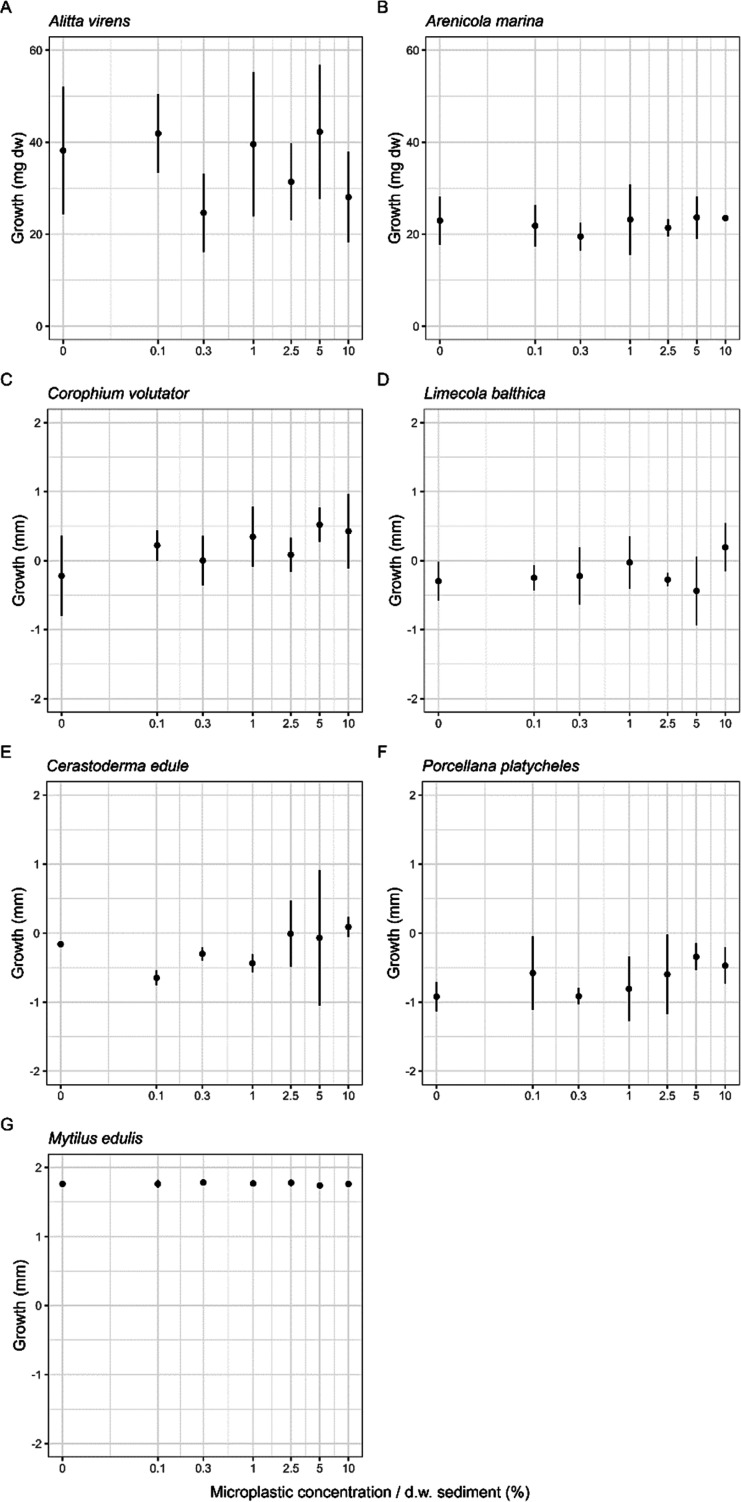
Mean growth (±sd) expressed in either length (mm) or weight (mg) of (A) *A. virens*, (B) *A. marina*, (C) *C. volutator*, (D) *L. balthica*, (E) *C. edule*, (F) *P. platycheles*, and (G) *M. edulis* exposed to ERMP + PS with concentrations up to 20% in dw sediment. No statistically significant effect thresholds were found. The exposure time for all marine species was 28 days. Note that concentrations are on a log scale. Additionally, the zero concentration has been converted to 0.01 to allow for plotting on the log scale.

The growth of marine and freshwater organisms was assessed using length after 28 days and the length of the population at the start. For the worm species *Tubifex* spp., *L. variegatus*, *A. virens*, and *A. marina*, growth is expressed in weight (mg of dw) and showed relatively high variability among replicates (Figures 3 and 4). As for the other organisms, *G. pulex*, *H. azteca*, *A. aquaticus*, *S. corneum*, *C. fluminalis*, *P. antipodarum*, *C. riparius*, *C. volutator*, *L. balthica*, *C. edule*, *P. platycheles*, and *M. edulis*, growth is expressed in length (mm) and variability among replicates was relatively low (Figures 3 and 4).

For the marine and freshwater species *H. azteca*, *A. aquaticus*, *S. corneum*, *C. fluminalis*, *P. antipodarum*, *Tubifex* spp., *C. riparius*, *A. virens*, *A. marina*, *C. volutator*, *C. edule*, *P. platycheles*, or *M. edulis*, no statistically significant effect thresholds were found after chronic exposure to ERMP with concentrations up to 10% dw in sediment for growth (Figures 3 and 4 and Tables S9 and S10 of the Supporting Information). For *L. balthica*, a significant effect threshold was found (*p* = 0.002); however, the dose–response curve was not significant, as also apparent by visual inspection (*p*_noEffect_ = 0.126). Additionally, for *G. pulex* exposed to PS fragments, a significant effect threshold was found (*p* = 0.006); however, the dose–response curve was not significant (*p*_noEffect_ = 0.332). For *G. pulex*, a reduction in growth was observed after chronic exposure to ERMP without PS fragment sediment (EC_50_ = 2.85 ± 1.39%; *p* = 0.050) with concentrations up to 20% in dw (Table S9 of the Supporting Information, Figure 3, and Table 1). Note that this effect threshold is not fit for regulatory purposes, because the control mortality was too high (40.9%). Additionally, for *L. variegatus*, a statistically significant adverse effect was found for growth (EC_50_ = 0.77 ± 0.14%; *p* = 0.007; *p*_noEffect_ = 2.32 × 10^3^; Table S9 of the Supporting Information, Figure 3, and Table 1). Finally, no effect threshold was found for the feeding rate of *G. pulex* after a 28 day exposure to PS fragments. For the feeding rate of *G. pulex* exposed to ERMP without PS fragments, a significant effect threshold was found (*p* = 2.16 × 10^–16^). However, the effect threshold concentration was not physically realistic; moreover, the dose–response curve was not significant (*p*_noEffect_ = 0.980), as also apparent by visual inspection (Table S9 and Figure S8 of the Supporting Information).

Our findings of *G. pulex* exposed to ERMP without PS are in accordance with the study done by Redondo-Hasselerharm et al.^23^ Also here, a reduction of growth on *G. pulex* was reported after exposure to PS, with a similar effect threshold (EC_50_ = 3.57%). As discussed previously, almost identical systematic approaches were used; however, in the present study, we used a more commonly occurring sediment with a lower TOM content and an even higher diversity of microplastic particles. The masking hypothesis previously mentioned does, however, not seem to apply to *G. pulex* from the study of Redondo-Hasselerharm et al.,^23^ suggesting a certain species-specific trait. Of all of the species tested and considering the results of the various tests done with *G. pulex*, this species seems to be the most sensitive to adverse effects of microplastics (Table 1).

For *L. variegatus* a significant negative effect was found for growth (EC_50_ = 0.77 ± 0.14%; *p* = 0.007). This is in contrast to the finding reported by other studies.^23,42^ For instance, Silva et al. reported on sub-organismal effects, such as depletion of energy reserves of *L. variegatus*; however, no adverse effects were found on biomass when exposed to only irregularly shaped PE. The differences in effects are likely due to the different types, shapes, sizes, and aging processes of the microplastics used. Again, to be conclusive about the latter, the ingestion of ERMP by *L. variegatus* would need to be studied.

No differences on feeding activity in the studies done for *G. pulex* (experiments 1 and 2) were found after a 28 day exposure to ERMP. This is similar to the results found in previous chronic exposure studies.^23,35^ Although testing solely PS and PET, respectively, these studies also did not find any effect on the feeding rate of *G. pulex*. These results show that the reduction in growth cannot be explained by the reduction in food uptake because this stays constant throughout treatment concentrations.^23^ Moreover, Straub et al. showed that, after chronic exposure to poly(methyl methacrylate) (PMMA) and polyhydroxy butyrate (PHB) in *Gammarus fossarum*, the assimilation efficiency and wet weight gain decreased; however, the feeding rate was not affected. It is likely that the decrease in growth can be explained by the mechanism inhibition of food assimilation and/or decreased nutritional value of food but also obstruction in the gut.^1,44^

### Different Responses to Microplastic Exposure for Marine versus Freshwater Species

After discussing the results at the single-species level, we look here and in the next section at differences between species categories. For instance, we tested whether the mortality response of marine species to an increasing dose of microplastics is different from that of the freshwater species. Interestingly, GLMM showed that the mortality risk for marine species decreases more rapidly as the concentration of microplastics increases compared to freshwater species (GLMM; *p* = 2.51 × 10^–5^; Table S11 of the Supporting Information). We consider these results to be preliminary. This is the first time that such a direct comparison between marine and freshwater species has been made, albeit with a limited amount of organisms, and more data should be considered to substantiate the detected trend. If further substantiated, such results are highly relevant for assessing risks using, for example, SSDs, often combining data on freshwater and marine species.^19^ A difference in the sensitivity between the habitats means that separate SSDs must be used. It is important to see whether the difference is present for all species or whether the significance level is dominated by a few species. In this regard, we mention the marine species *C. edule*. For this species, the mortality decreased with an increasing concentration, which partly determines the result for the entire group.

### Different Responses to Microplastic Exposure for Groups of Species with Different Feeding Traits

We also tested whether the responses of species to an increasing dose of microplastics are different for groups of species with different feeding traits. We distinguished four types of main feeding traits: filter feeders, sediment/deposit feeders, sediment grazers, and facultative deposit feeders (Table S12 of the Supporting Information). For mortality, no significant differences were found, except for the group of species feeding in the water column (filter feeders). Increasing exposure to microplastics has a greater effect on organisms exposed to water than on organisms in any of the other feeding guilds (GLMM; *p* < 3.90 × 10^–5^; parts a–d of Table S13 of the Supporting Information). Here too, *C. edule* falls within the deviant group, a species whose mortality decreases with increasing concentration. Again, we can only speculate about possible explanations for the aberrant exposure from the aqueous phase. One possible explanation is that species that feed on the water above the sediment have a higher general sensitivity to particulate matter than species that are naturally more closely associated with sediment because that is their natural habitat.

### Limitations and Recommendations for Future Work

Here, we have provided 17 dose–response relationships consisting of 5 effect threshold concentrations for 16 freshwater and marine benthic macroinvertebrate species using an environmentally relevant mixture of microplastic particles, demonstrating that this can be done under the strictest methods of quality assurance. Nevertheless, some species (*G. pulex*, *H. azteca*, *A. aquaticus*, *S. corneum*, and *C. edule*) exhibited high mortality rates in their control groups, rendering these results less suitable for regulatory considerations. Although these results have given us valuable insight, we suggest that these particular species be re-evaluated in future studies.

Earlier studies did not test such a wide range of species under the same conditions, were less reliable, and/or were less fit for the purpose concerning risk assessment.^1^ Rescaling and alignment methods have been developed to correct for differences among studies that target different microplastic size ranges, densities, or shape categories.^6,17^ However, the present effect data would require no or only a very limited level of corrections because microplastic particles were already close to those occurring in (aquatic) environmental samples. This enables direct use of the present data in risk assessment without the need for mathematical alignments. Furthermore, for the present microplastic mixture used, a probability density function (PDF) for size was provided, thus still allowing alignments to microplastic exposure conditions that would deviate from those represented by our tested mixture. We propose that PDFs are always provided when microplastic particles are used in tests, so that users can translate the test results into other conditions. Examples of how the results of these types of complex exposures can be applied to risk assessments have been outlined in recent literature.^19,24^

We did not find statistically significant effects on mortality or emergence for the freshwater and marine species *G. pulex*, *H. azteca*, *A. aquaticus*, *C. fluminalis*, *P. antipodarum*, *Tubifex* spp., *C. riparius*, *A. virens*, *L. balthica*, *C. volutator*, *A. marina*, *P. platycheles*, or *M. edulis* (Tables S7 and S8 of the Supporting Information). Additionally, chronic exposure to ERMP with concentrations up to 10% dw in sediment caused no significant effect on growth for the marine and freshwater species *H. azteca*, *A. aquaticus*, *S. corneum*, *C. fluminalis*, *P. antipodarum*, *Tubifex* spp., *C. riparius*, *A. virens*, *A. marina*, *C. volutator*, *L. balthica*, *C. edule*, *P. platycheles*, *M. edulis* (Tables S9 and S10 of the Supporting Information). Ideally, SSDs used for ecological risk assessment contain data on end points that are directly relevant to the population level, like reproduction, mortality, and growth. Here, we only considered such relevant end points, which implies that it is legitimate to speculate on implications for risks of microplastics in aquatic sediments based on our data. We detected the number concentration effect threshold EC_10_ for adverse effects for two freshwater species: 6.48 × 10^6^ number/kg (growth of *G. pulex* exposed to ERMP without PS) and 2.88 × 10^7^ and 1.12 × 10^8^ number/kg (growth and reproduction of *L. variegatus*). When these effect threshold concentrations were compared to rescaled measured environmental concentrations (MECs) for Liangfeng River sediments in China of 2.21 × 10^8^ number/kg of dw^26,24^ or for Menomonee River sediments in the U.S.A. of 1.73 × 10^8^ number/kg,^24,25^ it becomes apparent that risks would be indicated for *G. pulex* and *L. variegatus* and sufficiently similar species if our test conditions would be sufficiently representative of *in situ* exposure conditions.

Interestingly, we detected positive effects of microplastics on habitat quality for two species, *C. edule* and *S. corneum*, besides negative effects for several other species. The simultaneous existence of effect mechanisms that lead to a decline of the population density for some species and to a lesser decrease in population density for some other species has crucial implications for understanding the effects of microplastics on the community level. Previously, an analysis of community effects after long-term exposure to nano- and microplastics has revealed that the abundance of some species decreased, whereas the abundance of other species increased, indicating either direct or indirect positive effects on the species level.^45^ Another example of this is that the increase in microplastic concentrations in the oceans has reduced substrate limitation for oviposition for the pelagic insect *Halobates sericeus*,^46^ which also constitutes a positive effect on the population level. Additionally, Canniff and Hoang showed in an algal experiment that microplastics enhanced the growth of *Raphidocelis subcapitata* and suggested that, when ingested by daphnids, they could provide a possible food source.^47^ Recently, Amariei et al. reported a negative effect of microplastic ingestion by daphnids as a result of food dilution; however, a positive effect on the population was observed when that same microplastic was carrying a nutritious biofilm, which is the default situation in nature.^20^ Obviously, these observations should not be taken as a reason to scale down the problem of microplastic particles in the environment. However, a thorough understanding of multifarious effects, including negative as well as positive population effects, is required to be able to understand and model secondary effects on the food web and ecosystem level.^48^

## Supporting Information Available

Detailed description of the Materials and Methods (pages S2–S11), QA/QC criteria score (Table S1), plastic items collected in Biesbosch, Netherlands (Figure S1), microplastics produced from grinded plastic items (Figure S2), high-resolution pictures of PS fragments and ERMP (Figures S3 and S4), particle length distribution of ERMP with PS fragments (Figure S5), size distribution of PS fragments (Figure S6), overview of tested freshwater and marine benthic macroinvertebrates (Tables S2 and S3), microplastic analysis of background contamination (Table S4), mean water quality parameters of freshwater and marine experiments (Tables S5 and S6), verification of exposure concentrations using loss on ignition (LOI) (Figure S7), statistical results of dose–response models (Tables S7–S10), results of GLMM (Table S11, Table S12, and parts a–d of Table S13), and mean feeding rate of *G. pulex* (Figure S8) (PDF)
