# Supplementary material for: Microplastic Effect Tests Should Use a Standard Heterogeneous Mixture: Multifarious Impacts among 16 Benthic Invertebrate Species Detected under Ecologically Relevant Test Conditions
Source: Environ Sci Technol. 2023 Nov 22;57(48):19430–41. doi: 10.1021/acs.est.3c06829 (PMC10702443; doi:10.1021/acs.est.3c06829)
Supplement: Supplementary file 1 — es3c06829_si_001.pdf [file es3c06829_si_001.pdf]

Supporting Information

Microplastic effects tests should use a standard heterogeneous mixture: Multifarious impacts among sixteen benthic invertebrate species detected, under ecologically relevant test conditions

Vera N. de Ruijter[1], Matthias Hof[1], Petranta Kotorou[1], Jesse van Leeuwen[1], Martine J. van den Heuvel-Greve[2], Ivo Roessink[3], Albert A. Koelmans[1]

[1] Aquatic Ecology and Water Quality Management Group, Wageningen University, P.O. Box 47, 6700 AA Wageningen, the Netherlands

[2] Wageningen Marine Research, Wageningen University & Research, P.O. Box 77, 4400 AB Yerseke, the Netherlands

[3] Wageningen Environmental Research, Wageningen University & Research, P.O. Box 47, 6700 AA Wageningen, the Netherlands

Pages: 36

Tables: 16

Figures: 8

Detailed description of Material and Methods

Quality assurance and quality control

Background contamination was minimized by QA/QC according to de Ruijter et al. (2020); washing all materials and tools with Milli-Q water, wearing 100% cotton lab coats, and covering beakers with aluminium foil.[1] In order to quantify the contamination, experimental units containing solely DSW or filtered seawater were added during the experiments. Systems (n=34) were closed and stored after the 28 exposure time for further analysis.

Verification of exposure concentrations

Following Redondo-Hasselerharm et al. (2018), after 28 days, the actual exposure concentrations were verified for the 0, 5 and 10% concentrations (n=4) of the experiments with *A. aquaticus*, *H. azteca*, *C. edule* and *P. platycheles*, using the loss on ignition (LOI) method.[2] For each of these experiments, in quadruplicate, the 0, 5 and 10% concentrations were dried at 105°C for 24 hours and consequently heated at 550°C for at least 3 hours. As both organic matter and microplastics will burn at 550°C, their weight percentages can be calculated using the 0% concentration as a baseline for average OM content. In order to validate this LOI method, 5 crucibles were filled with approximately 1 g of the pure ERMP mix and burned at 550°C, showing an excellent recovery of 98.62% ± 0.054 (n=5). Results showed that for the experiments with *A. aquaticus*, *H. azteca* and *C. edule* 80 to 90% of the nominal concentration was maintained throughout the tests (Figure S7). As *P. platycheles* moved the sediment around quite a bit, 64 ± 5.4% of the nominal concentration was maintained in the sediment throughout the tests (Figure S7). That bioturbation can lead to partial exposure to ERMP via the aqueous phase for some organisms was considered part of the desired ecological relevance of the tests.

Verification of background contamination

During the microplastic exposure experiments, additional experimental units (n=34) were added with DSW alone or filtered seawater to quantify background contamination from atmospheric deposition, if any. Water samples (n=11) were filtered on a 20 µm aluminium filter whereafter rinsed and immersed in 15 ml H_20_2 for 48h at 37 °C. Consequently, the solution was filtered on an Anodisc (pore size 0.2 µm, Ø=25 mm, Whatman) and dried in an oven at 37 °C for at least 2 days. The Anodisc filters were placed on a calcium fluoride crystal window and analysed with a Cary 620 FT-IR Imaging Microscope with Focal Plane Array detector and 4x objective.[3]

FTIR data were analysed with Simple and MAPP software respectively.[4] 100-200 green spherical PE particles of ~ 90 µm were added to milli-Q in order to determine the recovery

(89.7% ± 4.5%, n=3). All handling took place in a laminar flow cabinet. While measures were taken to avoid contamination 26.8 ± 33.1 background particles were found per experimental unit (blank and recovery corrected). Background particle mass was calculated using particle-specific volume and polymer ID [4], which resulted in 3.14 (± 5.63) $\times 10^{-3}$ g/experimental unit (Table S4). This constitutes a negligible fraction of 1.6% of the lowest exposure concentration of 0.1% weight percentage.

Preparation of environmentally relevant microplastic particles

Microplastic particles with varying polymer type, size, shape and colour were created in the laboratory, in proportions that match those occurring in the environment as specified below.[1, 5] First, naturally aged macroplastic items such as bottles, buckets, packaging materials and rope were collected in the National park 'de Biesbosch', The Netherlands (Figure S1). The items were washed in a dishwasher and consequently analysed with ATR-FTIR (Agilent Cary 630) in order to determine polymer identity and purity, and sorted accordingly. Per polymer type (PE, PP, PET), plastic items were manually cut into pieces of approximately 0.5 to 1.0 cm, whereafter the fragments were grinded (Ultra Centrifugal Mill ZM 200) with a stainless steel 2 mm ring sieve (Figure S2). Liquid nitrogen was used in order to cool the mill and to prevent the plastics from melting. This process was repeated with half of the resulting particles with a ring sieve size of 1.0 and 0.5 mm respectively. With the exception of the PP particles which melted when using the 0.5 mm ring sieve size. Additionally it was not possible to mill the PP fibres or PET fragments with a 2.0 mm ring sieve as these melted even when an ample amount of liquid nitrogen was added.

Per polymer, microplastic size fractions < 0.5 mm, 0.5 – 2 mm and > 2mm were analysed separately. In order to illustrate the shapes of the different size fractions, high resolution pictures were made with an Olympus SZX10 stereomicroscope (Figure S3, 4). Particles were analysed for size (width, length, height, diameter) with Laser Direct Infra-Red spectroscopy (LDIR 8700, Agilent).[6] Smaller particles, resulting from grinding with a sieve size of 0.5 mm, were analysed with automatic particle characterization, with maximum particle sensitivity and a selected size range of 10 to 5000 µm. All resulting particle identifications were visually inspected in order to check for overlapping particles and false identifications. Particles which resulted from grinding with the 2.0 mm sieve were measured manually with the LDIR measurement tool. To enable weight to particle conversions, microplastic LDIR samples were accurately weighed before being measured and counted. The amount of particles per gram in the final mix was approximately 5.9×10^6. This translated to a particle concentration of 5.9×10^6, 1.8×10^7, 5.9×10^7, 1.5×10^8, 2.9×10^8, 5.9×10^8/kg dw soil for the weight concentrations of 0.1, 0.3, 1.0, 2.5, 5.0, 10.0%/dw sediment used in the bioassays.

After particle characterization of the separate size and polymer fractions, they were combined to form an environmentally relevant microplastic particle mixture, similar to relative abundances of polymers and sizes as found globally in the aquatic environment.[7] The weight distribution of polymers used to create the microplastic mix consisted of irregular PE fragments (34%), irregular PP fragments (15.9%), PP fibres (10.5%), and irregular PET fragments (20.6%). Additionally, irregular PS fragments (19%) were added to the mixture, which were acquired from Axalta Coating Systems GMBH, Cologne, Germany.[2] PS

polymer type was confirmed with FTIR and the size distribution was measured with a Mastersizer 3000 particle size analyser (Malvern Instruments). Additionally, high resolution pictures were made with an Olympus SZX10 stereomicroscope and analysed with ImageJ [8] for length and width of the PS particles. The reason for conducting an additional analysis based on laser diffraction (Mastersizer 3000) was that we wanted to be able to compare the PS powder with the PS powder used in our previous study (Redondo-Hasselerharm et al., ES&T, 2018). Note that laser diffraction assumes all particles are spheres and thus provides only equivalent sphere parameters. Such data cannot be directly compared with the PSD data reported by Kooi et al. (2021), which we used to mimic the power law slope for ERMP, as the latter data are based on particle length information from IR-based Image analysis. Therefore, LDIR data were used, as LDIR spectroscopy provides actual lengths, width, and shape (ir-)regularity data, which were fit for the purpose of our study.

In order to remove any additives present in the plastic, microplastics were washed with methanol and hexane three times and mixed on a shaker table for at least two hours per wash.[1,9] A 37 µm metal sieve was used to squeeze the methanol and hexane out of the particle mixture, after which particles were gently dried in a fume hood for two days. Note that contamination precautions at this stage were not needed because any addition from external sources would be minor and would only add to the desired diversity of the material, which was thoroughly characterised anyway.

Microplastic characterisation

The size distribution based on the particle distribution of the ERMP mix with PS ranged from 9 to 5386 µm in their longest dimension with a modus around 48 µm (Figure S5). The 75th percentile of the ERMP mix with PS particles is situated at 97 µm. The distribution of ERMP with PS was fitted to a power law $y = bx^{-\alpha}$, where α is the slope. For the complete ERMP mix, with PS, a slope of 3.28 ± 0.02 was measured which is equal to the slope found for microplastic particles in freshwater sediment (3.25 ± 0.19).[10,11] The mean slope is almost identical to microplastics found in freshwater sediments $\alpha = 3.25 \pm 0.19$.[11] The polymer types PET and PE that were grinded with a 0.5 mm sieve had a mean particle size of 202 µm and a median at 128 µm, with sizes ranging from 15 to 2245 µm. The PP and PE particles that were grinded with the 2.0 mm sieve had a mean of 281 µm and a median of 150 µm, with sizes ranging from 53 to 2165 µm. The PET particles grinded with a 2.0 mm sieve had a had a mean particle size of 2230 µm and a median of 2456 µm, with sizes ranging from 96 to 4588 µm. Microplastic fibres produced from PP rope had a mean length of 2012 µm and a mean width of 224 µm, with sizes ranging from 329 to 5386. The size distribution of PS MPs measured with ImageJ [8] ranged from 9 to 366 µm with a median of 67 µm and a mean of 78 µm. The size distribution of PS MPs measured with Mastersizer ranged from 14.5 to 400 µm with a modus centred at 32 µm (Figure S6).

Test organisms

G. pulex, *S. corneum* and *A. aquaticus* were collected from a pond (Sinderhoeve Renkum, The Netherlands). *P. antipodarum* were collected from a pond at the campus of Wageningen University and Research (Wageningen, The Netherlands). *L. variegatus* and *H. azteca* were obtained from a culture from Wageningen Environmental Research (Wageningen, The Netherlands). *Tubifex spp.* were obtained from a local pet shop. *C. fluminalis* was collected at Groene Heuvels (Ewijk, The Netherlands). Egg sacks of *C. riparius* were provided by the University of Amsterdam (The Netherlands). First instar larvae of *C. riparius* were used and transferred to the experimental units with glass pipets and petri dishes within 48 hours after hatching.

Marine species selected for this study were *Alitta Virens*, *Limecola balthica*, *Corophium volutator*, *Arenicola marina*, *Cerastoderma edule*, *Porcellana platycheles* and *Mytilus edulis* (Table S3). *A. virens* were obtained from live bait farmer Topsy Baits (Wilhelminadorp, The Netherlands) and *M. edulis* was collected at Brouwersdam (Ouddorp, The Netherlands). The other marine species were collected from the Eastern Scheldt, an estuary in the Netherlands, at the following sites: *L. balthica* and *C. edule* were collected at an intertidal flat near Roelshoek. *C. volutator* were collected at a mudflat in the Oesterput. *A. marina* were collected at intertidal flat next to the Oesterdam. *P. platycheles* were collected from intertidal flat at Goese Sas. Freshwater and marine test organisms were obtained from non-polluted locations and were acclimatized for at least 2 days at 16 ± 1 °C, in DSW (freshwater species) or filtered seawater (marine species) before the start of the experiment.

Sediment

Sediments were sieved with 2 mm sieve and stored at -20°C in order to preserve organic matter and kill any organisms present. A subsample was set aside in order to analyse background contamination. Prior to use in the experiments, sediment was thawed, the top layer of water disposed, homogenized and total organic matter content (TOM) was analysed as loss on ignition. The freshwater sediment had a TOM content of 6.8% ± 0.42 (n=5). The marine sediment had a TOM content of 3.8% ± 0.16 (n=10).

Experimental set up

In total 17 chronic, single species bioassays were performed. The systematic testing approach in this study is similar to the one followed by Redondo-Hasselerharm *et al.* (2018), however in the previous study sediment had a TOM content of 31.6% ± 3.5 (n=4).[2] While ecologically justifiable, this high TOM content could possibly mask adverse effect of microplastics, and hence we chose a sediment with a lower, more common TOM content. In order to maintain comparability between our systematic approaches, we repeated the previous experiment by Redondo-Hasselerharm *et al.* (2018) with *G. pulex.* Once with the lower TOM content sediment and PS fragments as used by Redondo-Hasselerharm, and once with the lower TOM content and with ERMP instead of PS fragments. For experiments 1 and 2, experimental units were made by either adding PS fragments or ERMP without PS-fragments to sediment (Table S2, 3) in the following concentrations 0, 0.5, 1, 3, 5, 10 and 20 weight %. For experiments 3 t/m 17 ERMP including PS fragments (Table S2, 3) were added to the sediment in the following concentrations 0, 0.1, 0.3, 1.0, 2.5, 5.0 and 10.0 weight %. Concentrations ranging from environmentally relevant (0 to 1.0%) to high concentrations (2.5-20%) are included in order to cover criteria related to relevance as well as to statistical rigour in finding an effect threshold.[1, 12] Sediment-microplastic mixtures were thoroughly, manually homogenized with a stainless steel spoon after which DSW or filtered seawater was gently added with a 3:1 water to sediment ratio. This ratio has been demonstrated to provide good water quality and habitat conditions during chronic exposures in earlier tests.[2, 12] Each experimental unit was made in quadruplicate and additionally four blanks (containing only DSW or seawater) were added in order to measure background microplastic contamination, e.g. from atmospheric composition. Systems were randomized and subsequently left to acclimatize for two weeks before adding the organisms. To each experimental unit, 11 to 22 organisms were added depending on the size of the organisms (Table S2, 3). Experimental units of *C. riparius* were covered with a 1 mm mesh after seven days to retain emerged adults. Freshwater organisms, excluding the crustaceans, were fed weekly with 0.1g/bioassay organic nettle powder dissolved in DSW. In contrast, the crustaceans *G. pulex*, *H. azteca* and *A. aquaticus* were fed dried poplar leaves. Marine filter feeders were fed weekly with 20 ml high density algal solution (*Halamphora coffeaeformis*). A*. virens* was fed 5.6% of its wet weight feed pellets supplied by Topsy Baits (Wilhelminadorp, The Netherlands). *C. volutator* was fed ground JBL Novo Prawn. Exposure duration for all organisms was 28 days. Dissolved oxygen, pH, temperature, conductivity/salinity and NH_3 concentrations were measured twice a week. In order to keep water quality parameters optimal, DSW and seawater were refreshed every other day (Table S5, 6).

Data analysis

Single species effect data

Selection of best dose-response model and assessment of threshold effect parameters

All single species dose-response data was analysed using the dose response curve (*drc*) package in R.[13] This package contains a range of models including 2 to 4 parameter log-logistic models and Weibull models. For the continuous response data growth, reproduction and feeding rate, log-logistic models with a Gaussian distribution were fitted. In order to evaluate the quality of the model fit, the normality of residuals were tested with the Shapiro-Wilk test and visually inspected with a Q-Q plot. The homogeneity of variance was checked with Levene's test. If the assumption of homogeneity of variance was not satisfied, robust standard errors were provided (*package lmtest*).[13, 14] For the endpoints mortality and emergence a log-logistic model with a binomial distribution was fitted. The best fitting model was selected based on the lowest AIC (*mselect function R*) [13] and visual inspection. The best fitting type of dose-response model, and corresponding threshold effect concentrations are reported (Table S7, S8, S9, S10).

Statistical significance of dose-dependency

For the best fitting model from the previous step, the selected best fitting dose-response model was compared to a linear regression model with a slope of 0, which corresponds to absence of a dose-response relationship. This was done using the log-likelihood ratio test (*noEffect function in R*) [13]. If the p-value of the log-likelihood ratio test ($p_{noEffect}$) is less than p= 0.05, the null hypothesis of 'no difference' was rejected and we concluded that there is a significant difference between the two models, implying that the dose-response relationship is statistically significant. If the p-value of the log-likelihood ratio test ($p_{noEffect}$) was > 0.05, we concluded, in combination with visual inspection, that the dose-effect relationship was not significant. The p- values of the log-likelihood ratio tests are reported for all endpoints and organisms (Table S7, S8, S9, S10).

Testing for differences between effects on freshwater versus marine species, and for differences between feeding traits

In order to explore if the effects of microplastics on mortality were different for species from the freshwater vs marine environment, a Generalized Linear Mixed model (GLMM) was used (*lme4 package R*).[15] As mortality is the response variable, the model was fitted using the binomial family with a logit link. The explanatory variables 'concentration' and 'environment'

were added as an interaction term. In order to allow for the variability between organisms, a random effect for the different organisms was added.

Similarly, a Generalized Linear Mixed model (GLMM) was used to explore if the effects of microplastics on mortality were different for the different feeding traits; filter feeders, sediment/deposit feeders, sediment grazers and facultative deposit feeders (Table S12). As mortality is the response variable, the model was fitted using the binomial family with a logit link. The explanatory variables ‘concentration’ and ‘feeding trait’ were added as an interaction term. In order to allow for the variability between organisms a random effect for the different organisms was added. The results of the GLMMs were interpreted by examining the estimated coefficients of the interaction term and accompanying standard error and p-value. These are provided for each GLMM. All statistical analyses and graphs were performed in RStudio.[16]

Table S1. Quality Assurance/Quality control (QA/QC) criteria score for testing effects of MP in aquatic test systems [1]

Criteria	Score (0-2)[a)]	Score explanation
**Particle Characterization**		
1. Particle size	2	Min/max size values and average size given (15 - 5386 µm) and particle size distribution measured and reported.
2. Particle shape	2	High resolution pictures of used microplastic mix are provided.
3. Polymer type	2	Polymer types (PS, PP, PE and PET) were identified with FTIR.
4. Source of MP	2	Source of environmental plastic (Biesbosch, the Netherlands) and description how MP was made in lab provided and source PS fragments (Axalta Coating Systems GMBH, Cologne, Germany) reported.
5. Data reporting	2	MP concentrations were reported as mass and particles amount.
**Experimental design**		
6. Chemical purity	2	To remove additives present, if any, the MP were washed with methanol and hexane three times.
7. Laboratory preparation	2	All materials used were washed with Milli-Q water, and non-plastic materials were used whenever possible. Bioassays were covered with aluminium foil to prevent contamination from air.
8. Verification of background contamination	2	Additional blank experimental units were used to determine background contamination .
9. Verification of exposure	2	MP exposure was verified with loss of ignition in 4 representative bioassays.
10. Homogeneity of exposure	2	Method of obtaining homogenous exposure (stirring) was described.
11. Exposure assessment	2	Organisms and egestion samples were taken to determine exposure[b)]
12. Replication	2	4 replicates were used.
**Applicable for Risk assessment**		
13. Endpoints	2	Ecologically relevant endpoints (survival, growth, reproduction) for risk assessments at the individual level were used.
14. Presence of natural (food) particles	2	Natural particles and food were added to test systems
15. Reporting of effect thresholds	2	Effect thresholds were reported as EC_{50} and EC_{10} with standard errors reported.
16. Quality of dose-response relationship	2	At least 7 concentrations including control
**Ecological relevance**		
17. Concentration range tested	2	2 environmentally relevant concentrations were used and motivated from measured environmental concentrations.
18. Aging and biofouling	1	Aged plastic was used. A biofilm was allowed to form during acclimatization, however, the biofilm was not characterized. Consequently only 1 point was given .
19. Diversity of MP tested	2	A variety of MP shapes and sizes was used.
20. Exposure time	2	The exposure time was 28 days.
**Total**	39 (97.5%)	

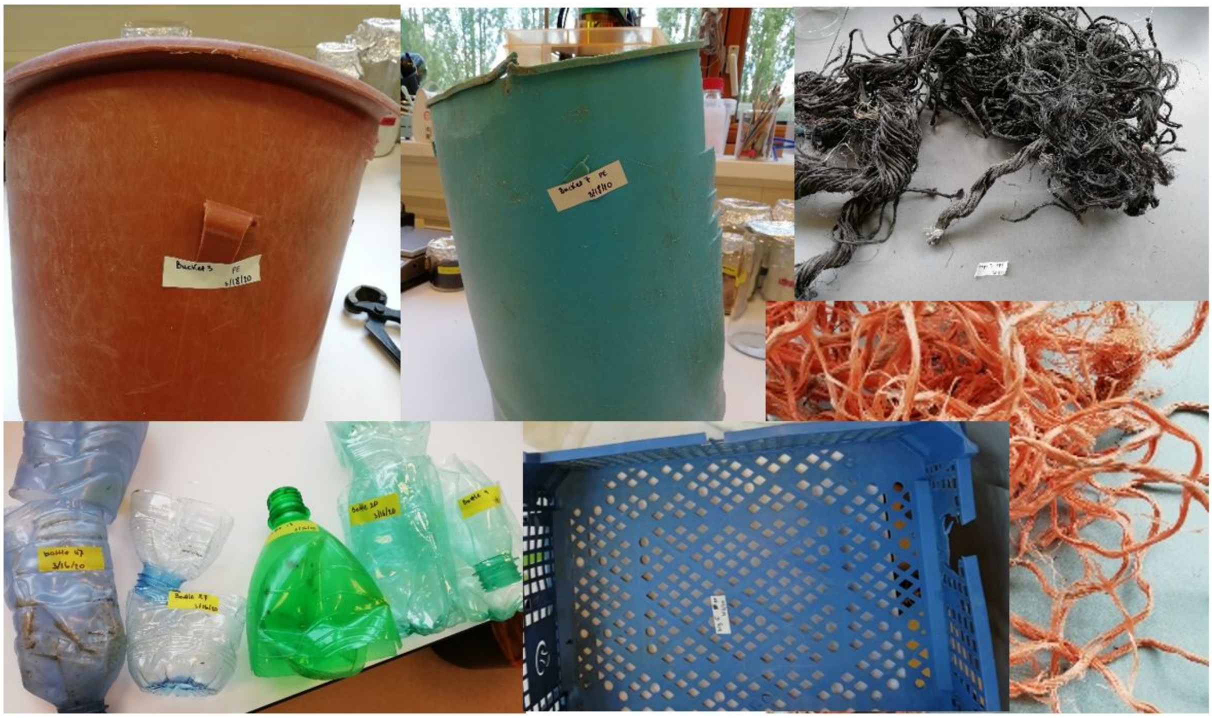

Figure S1. Variety of plastic items collected in the Biesbosch, The Netherlands. Starting left upper corner, clockwise: Brown PE bucket, blue PE bucket, black PP rope, orange PP rope, blue PP crate, green, blue and transparent PET bottles.

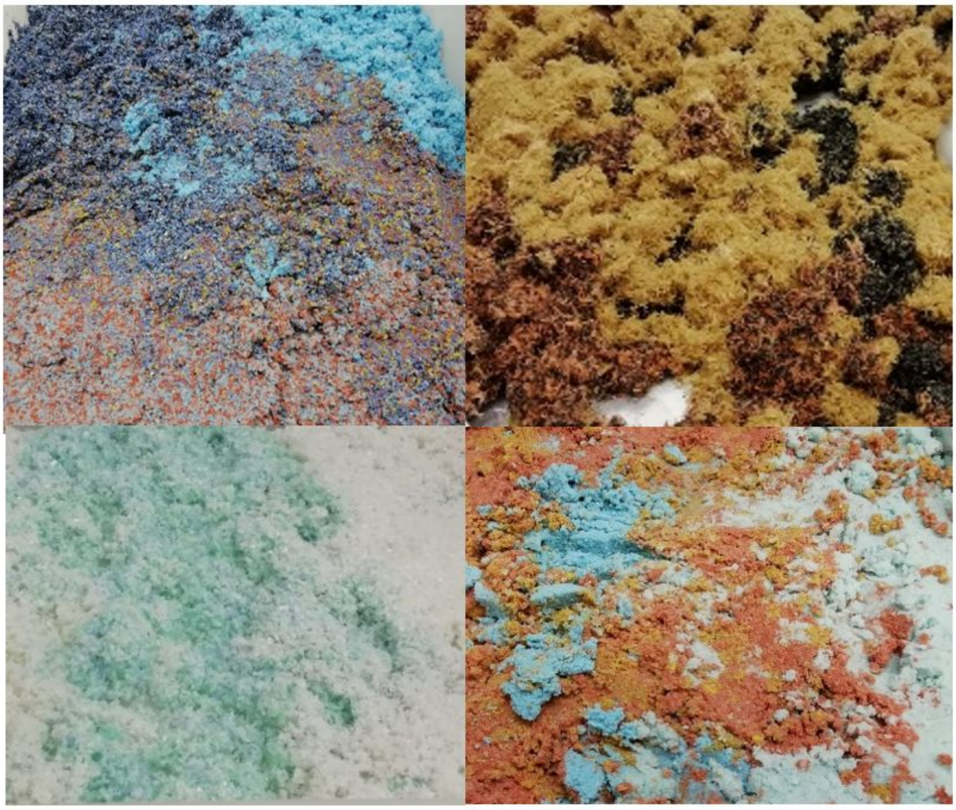

Figure S2. Microplastics from grinded plastic items. Starting left upper corner; clockwise: irregular PE fragments , PP fibres, irregular PP fragments and irregular PET fragments.

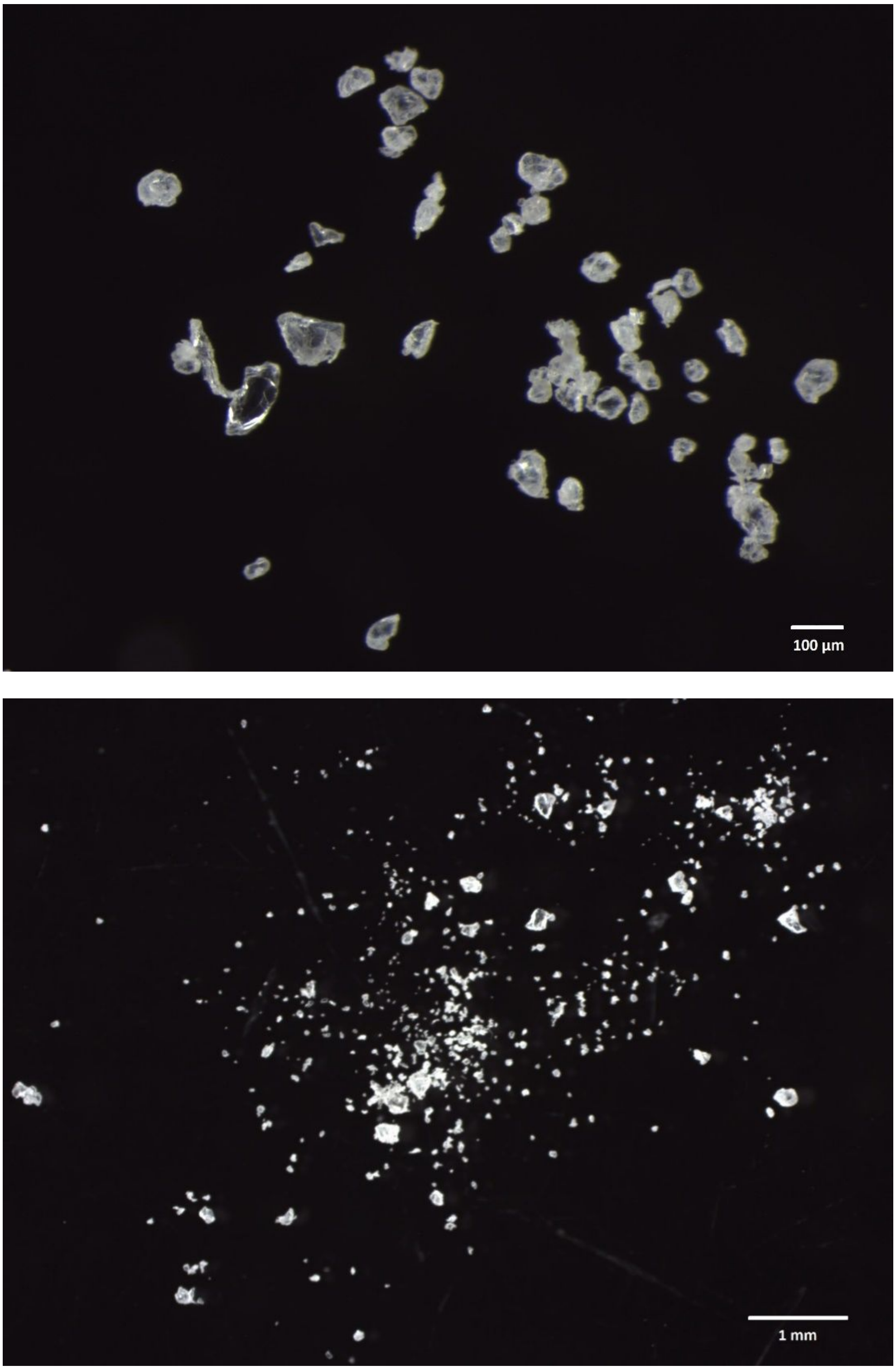

Figure S3. High resolution pictures of PS-fragments made with an Olympus SZX10 stereomicroscope.

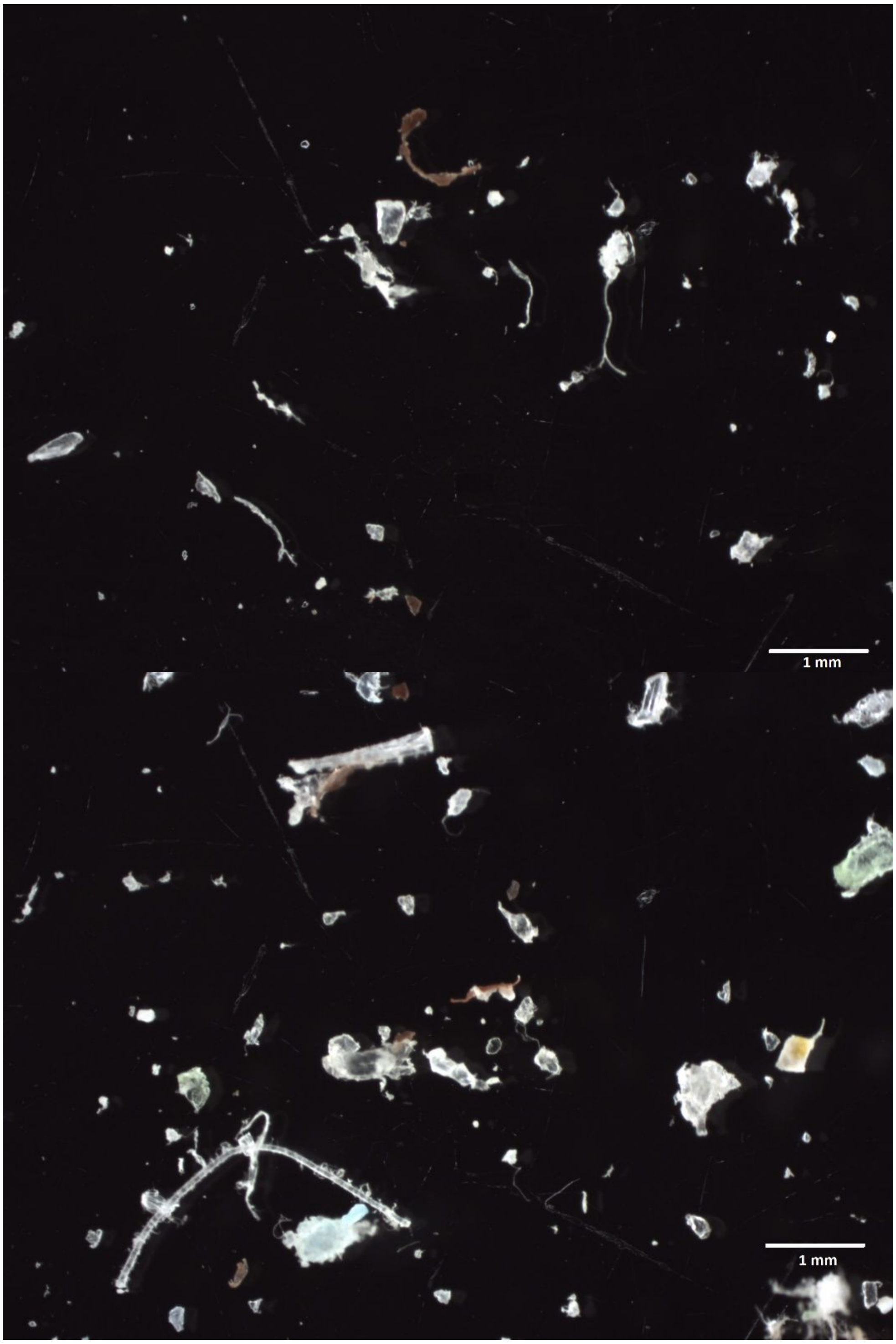

Figure S4. High resolution pictures of environmentally relevant microplastics mix made with an Olympus SZX10 stereomicroscope.

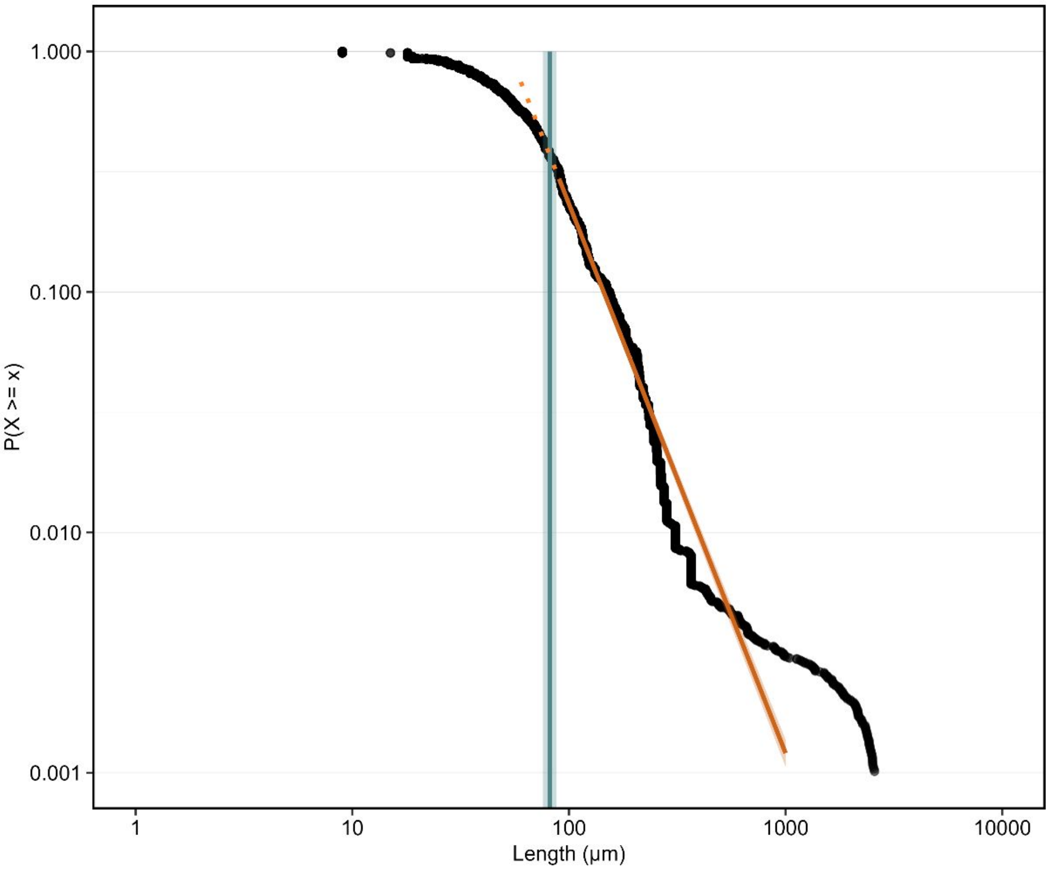

Figure S5. Particle length distribution of ERMP with PS fragments. Measurements were made with an Agilent LDIR 8700 Imaging system. The distribution was fitted to a power law $y = bx^{-\alpha}$, where α is the slope indicated by the orange line: α = 3.28 ± 0.02.[10, 11] The slope α was designed to be equal to the slope found for microplastic mixtures in freshwater sediments, which was reported earlier as α = 3.25 ± 0.19.[11]

B

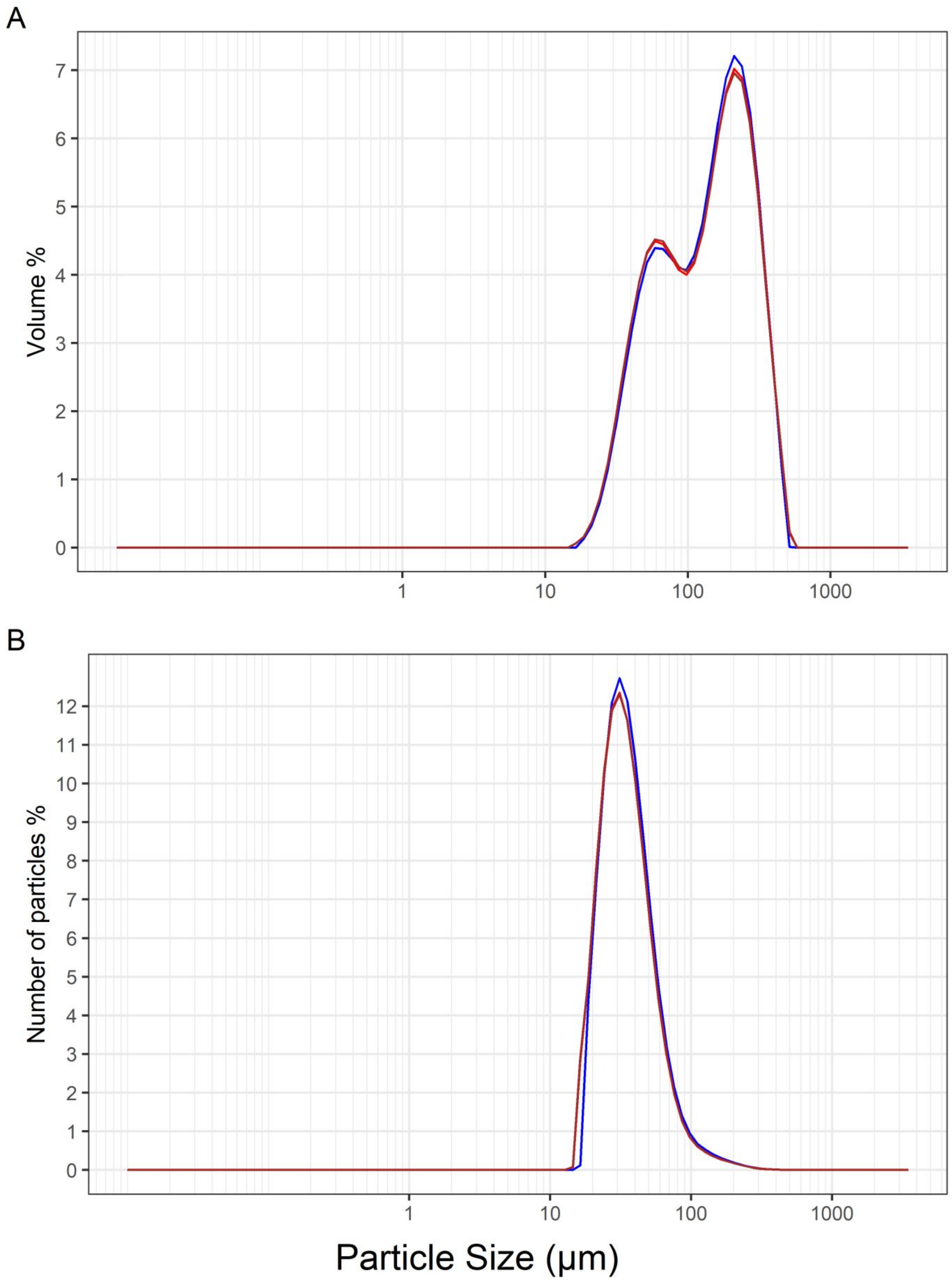

Figure S6. Size distribution of PS-fragments (A) based on volume of particles and (B) based on number of particles. Measurements made with a Mastersizer 3000 (Malvern Instruments) (n = 5).

Table S2. Overview of endpoints for the tested benthic freshwater macroinvertebrates.

# Exp.	Species	Feeding behaviour	Endpoints	MP tested	Start		Mortality control
					#	Weight/length	
1	*Gammarus pulex* (amphipod)	Epibenthic species, that is mainly regarded as a shredder.	Mortality, growth, feeding rate	PS	11	5.87 ± 0.07 mm	15.9 ± 11.8 %
2	*Gammarus pulex* (amphipod)	Epibenthic species, that is mainly regarded as a shredder.	Mortality, growth, feeding rate	ERMP	11	5.79 ± 0.11 mm	40.9 ± 10.2 %
3	*Hyalella azteca* (amphipod)	Epibenthic species, that is mainly regarded as a shredder.	Mortality, growth, feeding rate	ERMP + PS	11	4.09 ± 0.66 mm	29.5 ± 7.5 %
4	*Asellus aquaticus* (waterlouse)	Epibenthic species that is mainly regarded as a shredder.	Mortality, growth, feeding rate	ERMP + PS	11	4.64 ± 0.77 mm	31.8 ± 10.2 %
5	*Sphaerium corneum* (clam)	Endo- and epibenthic species that filters surrounding water for nutrients and also siphons the sediment top layer for organic matter.	Mortality, growth	ERMP + PS	11	4.11 ± 0.60 mm	34.1 ± 16.2 %
6	*Corbicula fluminalis* (clam)	Endo- and epibenthic species that	Mortality, growth	ERMP + PS	11	4.10 ± 0.58 mm	0.0 ± 0.0 %

		filters surrounding water for nutrients and also siphons the sediment top layer for organic matter.					
7	*Potamopyrgus Antipodarum* (mud snail)	Epibenthic species, nocturnal grazer-scraper, that feed on plant and animal detritus.	Mortality, growth	ERMP + PS	20	2.69 ± 0.06 mm	7.5 ± 5.6 %
8	*Tubifex* spp. (worm)	Predominantly sediment-dwellers, decompose sediment.	Mortality, growth	ERMP + PS	20	1.93 mg	6.3 ± 10.8 %
9	*Lumbriculus variegatus (worm)*	Predominantly sediment-dwellers, decompose sediment.	Reproduction, population growth	ERMP + PS	20	16.48 ± 5.53 mg	2.9 ± 0.1 [a]
10	*Chironomus riparius* (midge)	Chironomid larvae are benthic species, nonselective deposit feeder.	Emergence	ERMP + PS	15	n.a.	40.0 ± 24.5 % [b]

[a] mean reproduction factor, [b] mean total not emerged. PS = polystyrene fragments, ERMP = environmentally relevant microplastics. # number of organism per test system. Starting weight and length ± standard deviation (s.d.)

Table S3. Overview of the tested benthic marine macroinvertebrates.

# Exp.	Species	Feeding behaviour	Endpoints	MP tested	Start		Mortality control
					#	Weight/length	
1	*Alitta Virens*	Epibenthic species, omnivorous	Mortality, growth	ERMP + PS	11	112.51 ± 3.35 mg	18.2 %
2	*Limecola balthica*	Endo- and epibenthic species, active suspension feeder and surface deposit feeder.	Mortality, growth, burrowing time	ERMP + PS	11	9.23 ± 0.16 mm	2.3 %
3	*Corophium volutator*	Endobenthic species, that eats organic matter. Surface deposit feeder, Active suspension feeder.	Mortality, growth	ERMP + PS	11	7.49 ± 0.25 mm	13.6 %
4	*Arenicola marina*	Sub-surface deposit feeder, Surface deposit feeder.	Mortality, growth	ERMP + PS	11	n.a.	20.5 %
5	*Cerastoderma edule*	Endo- and epibenthic species, active suspension feeder.	Mortality, growth	ERMP + PS	11	14.20 ± 0.51 mm	75.0 %

6	*Porcellana platycheles*	Epibenthic species, filter feeder.	Mortality, growth	ERMP + PS	11	8.17 ± 0.52 mm	0.0 %
7	*Mytilus edulis*	Endo- and epibenthic species, active suspension feeder.	Mortality, growth	ERMP + PS	11	15.53 ± 0.31 mm	4.5 %

PS = polystyrene fragments, ERMP = environmentally relevant microplastics. # number of organism per test system. Starting weight and length ± standard deviation (s.d.)

Table S4. MP analysis of background contamination samples (n=11).

# microplastics /experimental unit	# microplastics /experimental unit corrected*	microplastics g/ experimental unit	microplastics g/ experimental unit corrected*
192 ± 145.3	26.8 ± 33.1	2.82E-3 ± 5.05E-3	3.14E-3 ± 5.63E-3

* samples were corrected with blanks (n=2) based on polymertype, shape, length and width.
Additionally samples were corrected for recovery = 89.7% ± 4.5% , n=3.

Table S5. Mean water quality parameters of freshwater experiments (Mean ± s.d.)

# Exp.	Species	T (°C)	pH (-)	EC (µS/cm)	O_2 (%)	NH_3 (mg N/L)
1	*Gammarus pulex*	16.4±0.2	8.1±0.3	791±132	99.7±1.8	0.09±0.16
2	*Gammarus pulex*	16.5±0.3	8.2±0.3	695±53	100.8±2.3	2.63±2.54
3	*Sphaerium corneum*	16.6±0.1	8.1±0.2	660±44	91.8±6.2	0.20±0.13
4	*Lumbriculus variegatus*	16.6±0.1	8.1±0.3	648±23	92.4±6.0	0.22±0.17
5	*Chironomus riparius*	16.5±0.1	7.94±0.1	580±24	88.7±4.8	0.07±0.02
6	*Potamopyrgus Antipodarum*	16.1±0.1	7.99±0.1	583±27	90.9±4.7	0.13±0.09
7	*Hyalella azteca*	15.7±0.2	8.0±0.2	641±25	99.7±4.3	2.24±2.46
8	*Asellus aquaticus*	15.7±0.4	8.0±0.1	622±24	110.3±5.6	2.57±2.60
9	*Tubifex* spp.	15.8±0.2	7.9±0.3	690±25	9.87±0.44	3.00±2.90
10	*Corbicula fluminalis*	15.8±0.4	8.0±0.1	684±27	99.4±8.0	2.36±2.24

Table S6. Mean water quality parameters of marine experiments (Mean ± s.d.)

# Exp.	Species	T (°C)	pH (-)	Salinity (ppt)	O_2 (%)
1	*Alitta Virens*	16.7±0.5	8.1±0.1	33.0±2.8	8.3±0.9
2	*Limecola balthica*	16.6±0.4	8.3±0.1	33.2±0.9	8.9±0.7
3	*Corophium volutator*	16.6±0.5	8.3±0.2	33.4±1.4	9.0±0.5
4	*Arenicola marina*	16.4±0.2	8.3±0.1	32.4±0.4	9.5±0.04
5	*Cerastoderma edule*	16.5±0.2	8.3±0.2	32.7±0.4	9.5±0.1
6	*Porcellana platycheles*	16.5±0.1	8.3±0.3	32.6±0.4	9.5±0.04
7	*Mytilus edulis*	16.9±0.2	8.1±0.1	33.4±0.7	9.5±0.1

Feeding rate equation

$$FR = \frac{(L1 \times Cl) - L2}{(Li1 + Li2) \times t/2} \quad (1)$$

The feeding rate (mg dw leaf/organism/d) of *G. pulex* was calculated from the loss of poplar leaves. Where L1 and L2 are the initial and final dry weight of poplar leaves respectively. Cl is the decomposition factor measured in the control systems. Li1 and Li2 are the initial and final amount of organisms in the system and t is the exposure time.

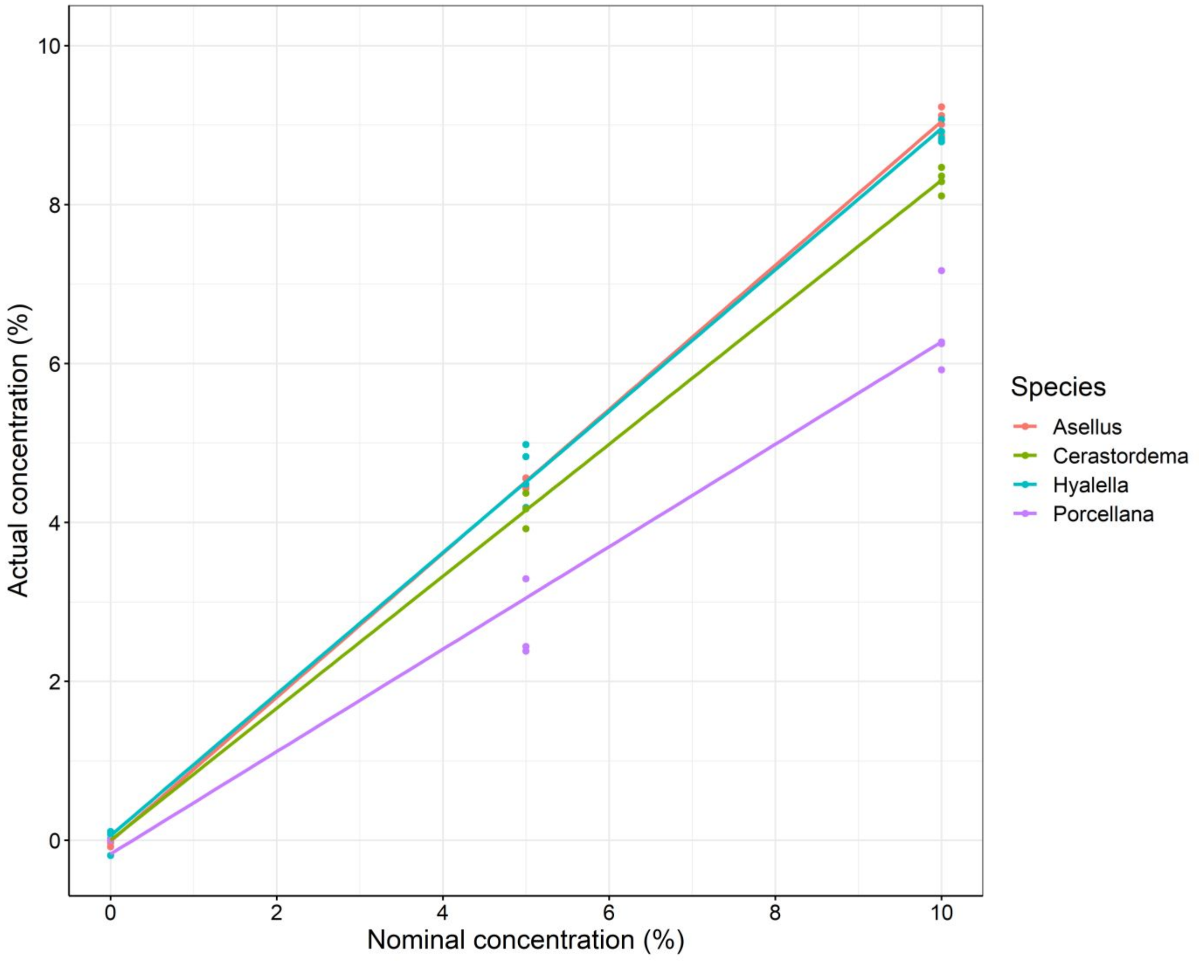

Figure S7. Verification of exposure concentrations using loss on ignition (LOI) at end of exposure for experiments with *A. aquaticus*, *H. azteca*, *C. edule* and *P. platycheles*.

Table S7. Estimates and statistical significance of effect threshold concentrations and best-fitting dose-response models for mortality freshwater species.

# Exp.	Species	Response variable	Distribution	Model	Estimate effect threshold ± std. error (% d.w.)	Estimate effect threshold p-value	Log-likelihood ratio test p-value (noEffect)
1	*Gammarus pulex (PS)*	Mortality	Binomial	LL.3	19.65 ± 8.12	**0.015***	0.311
2	*Gammarus pulex (ERMP-PS)*	Mortality	Binomial	W1.3	1.29E+03 ± 4.71E+04	0.978	1.000
3	*Hyalella azteca*	Mortality	Binomial	W1.3	0.02 ± 0.62	0.975	1.000
4	*Asellus aquaticus*	Mortality	Binomial	W1.4	NA	NA	NA
5	*Sphaerium corneum*	Mortality	Binomial	LL.3	9.88 ± 0.68	**2E-16*****	**0.013***
6	*Corbicula fluminalis*	Mortality	Binomial	W1.3	0.10 ± 0.11	0.381	0.072
7	*Potamopyrgus Antipodarum*	Mortality	Binomial	W1.3	2.89E+03 ± 8.21E+04	0.972	1.000
8	*Tubifex* spp.	Mortality	Binomial	W1.3	0.80 ± 30.87	0.979	1.000
9	*Lumbriculus variegatus*	Reproduction factor	Normal	W1.4	2.67 ± 0.76	**0.002****	**0.009****
10	*Chironomus riparius*	Emergence	Binomial	W2.4	0.22 ± 0.07	**0.003****	0.341

Significant differences ($p < 0.05$) between the dose-dependent and the dose-independent model are highlighted in bold.
Dose-response curve package in R provides the following models: Weibull type I model (W1.x) and log logistic (LL.x), with x giving the number of parameters fitted.

Table S8. Estimates and statistical significance of effect threshold concentrations and best-fitting dose-response models for mortality marine species

# Exp.	Species	Response variable	Distribution	Model	Estimate effect threshold ± std. error (% d.w.)	Estimate effect threshold p-value	Log-likelihood ratio test p-value (noEffect)
1	*Alitta Virens*	Mortality	Binomial	LL.2	NA	0.030	1.00
2	*Arenicola marina*	Mortality	Binomial	W1.4	NA	0.190	1.00
3	*Corophium volutator*	Mortality	Binomial	LL.2	5.40E+03 ± 1.30E+04	0.678	1.00
4	*Limecola balthica*	Mortality	Binomial	LL.2	1.83E+05 ± 1.47E+06	0.901	1.00
5	*Cerastoderma edule*	Mortality	Binomial	W1.3	2.14 ± 0.71	**0.003****	**3.22E-15*****
6	*Porcellana platycheles*	Mortality	Binomial	NA	1.82E+03	-	-
7	*Mytilus edulis*	Mortality	Binomial	NA	-	-	-

Significant differences (p< 0.05) between the dose-dependent and the dose-independent model are highlighted in bold. Dose-response curve package in R provides the following models: Weibull type I model (W1.x) and log logistic (LL.x), with x giving the number of parameters fitted.

Table S9. Estimates and statistical significance of effect threshold concentrations and best-fitting dose-response models for growth freshwater species

# Exp.	Species	Response variable	Distribution	Model	Estimate effect threshold ± std. error (% d.w.)	Estimate effect threshold p-value	Log-likelihood ratio test p-value (noEffect)
1	*Gammarus pulex (PS)*	Growth	Normal	W1.4	5.06 ± 1.68	**0.006****	0.332
1	*Gammarus pulex (PS)*	Feeding rate	Normal	LL.2	1.31E+03 ± 1.47E+05	0.993	1.000
2	*Gammarus pulex (ERMP - PS)*	Growth	Normal	LL.2	2.85 ± 1.39	**0.050***	**6.28E-04*****
2	*Gammarus pulex (ERMP - PS)*	Feeding rate	Normal	LL.4	1.59E+27 ± 10	**2.2E-16*****	0.980
3	*Hyalella azteca*	Growth	Normal	W2.4	0.80 ± 0.68	0.251	0.154
4	*Asellus aquaticus*	Growth	Normal	W2.4	0.01	NA	0.929
5	*Sphaerium corneum*	Growth	Normal	LL.4	2.24 ± 1.11	0.055	0.300
6	*Corbicula fluminalis*	Growth	Normal	LL.3	0.11 ± 0.18	0.546	0.149
7	*Potamopyrgus Antipodarum*	Growth	Normal	LL.3	0.09 ± 0.995	0.995	0.290
8	*Tubifex* spp.	Growth	Normal	LL.4	2.10 ± 0.998	0.998	0.511
9	*Lumbriculus variegatus*	Growth	Normal, possible heteroskedasticity	W1.4	0.83 ± 0.28	**0.007****	**2.32E-03****
10	*Chironomus riparius*	Development	Normal	W1.3	1.56E+03 ± 2.78E+03	0.581	0.844

Significant differences ($p < 0.05$) between the dose-dependent and the dose-independent model are highlighted in bold.. Dose-response curve package in R provides the following models: Weibull type I model (W1.x) and log logistic (LL.x), with x giving the number of parameters fitted.

Table S10. Estimates and statistical significance of effect threshold concentrations and best-fitting dose-response models for growth Marine species.

# Exp.	Species	Response variable	Distribution	Model	Estimate effect threshold ± std. error (% d.w.)	Estimate effect threshold p-value	Log-likelihood ratio test p-value (noEffect)
1	*Alitta Virens*	Growth	Normal	W2.4	0.12 ± 0.37	0.745	0.593
2	*Arenicola marina*	Growth	Normal	LL.3	234.49 ± 7271.41	0.975	1.000
3	*Corophium volutator*	Growth	Normal	LL.3	18.12 ± 145.97	0.902	0.053
4	*Limecola balthica*	Growth	Normal, possible heteroskedasticity	LL.3	7.11 ± 2.09	**0.002****	0.126
5	*Cerastoderma edule*	Growth	Normal	LL.3	1.75 ± 2.86	0.549	0.080
6	*Porcellana platycheles*	Growth	Normal	W1.3	21.69 ± 41.82	0.609	0.103
7	*Mytilus edulis*	Growth	Normal	LL.4	3.70 ± 1.82	0.056	0.497

Significant differences ($p < 0.05$) between the dose-dependent and the dose-independent model are highlighted in bold. Dose-response curve package in R provides the following models: Weibull type I model (W1.x) and log logistic (LL.x), with x giving the number of parameters fitted.

Table S11. Estimated coefficients of the Generalized Linear Mixed Model with binomial distribution, with response variable Mortality, and explanatory variables Concentration and Environment (Marine and Freshwater).

Coefficients:	Estimate	Std. Error	z value	Pr(>|z|)
Intercept	-1.335	0.320	-4.170	3.05E-05 *** [a]
Concentration	-0.253E-03	0.013	-0.020	0.984[b]
Environment Freshwater vs Marine	-0.415	0.472	-0.880	0.379[c]
Interaction Concentration Freshwater vs Marine	-0.108	0.026	-4.214	**2.51E-05 ***[d]**

Significant findings are ($p < 0.5$) are highlighted in bold. Significant codes: <0.0001 '***', < 0.001, '**' < 0.01, '*' < 0.05.The organisms *Lumbriculus variegatus* and *Chironomus riparius* were excluded from analysis as they have different endpoints, reproduction and emergence, respectively.

[a] Baseline.

[b] The variable 'concentration' alone does not appear to be a significant predictor of mortality.

[c] The variable 'environment' alone does not appear to be a significant predictor of mortality.

[d] The interaction term indicates whether the mortality of marine or freshwater organisms is more affected by the concentration of microplastics, which is statistically significant.

Table S12. Division of organisms with different feeding traits.

**Filter feeders**	**Sediment/deposit feeder**	**Sediment grazer/scavengers**	**Facultative deposit feeders**
Porcellana platycheles	*Arenicola marina*	*Gammarus pulex (PS)*	*Limecola balthica*
Mytilus edulis	*Alitta virens*	*Gammarus pulex (ERMP-PS)*	*Sphaerium corneum*
Cerastoderma edule	*Potamopyrgus antipodarum*	*Hyalella azteca*	*Corbicula fluminalis*
	Tubifex spp.	*Asellus aquaticus*	*Corophium volutator*

Table S13a. Estimated coefficients of the Generalized Linear Mixed Model with binomial distribution, with response variable Mortality, and explanatory variables ‘Concentration’ and “Feeding trait” with four levels; Filter feeders, Sediment/deposit feeder, Sediment grazer/scavengers, Facultative deposit feeders, see table S12. The group Sediment/deposit feeder is used as a reference to compare the other groups.

Coefficients:	Estimate	Std. Error	z value	Pr(>|z|)
Intercept	-1.671	0.386	-4.336	1.45E-05 *** [a)]
Concentration	0.005	0.017	0.261	0.794 [b)]
Trait Sediment grazer/scavengers	0.774	0.627	1.234	0.217 [c)]
Trait Facultative deposit feeders	-0.504	0.588	-0.857	0.391 [c)]
Trait Filter feeders	0.518	0.631	0.821	0.412 [c)]
Concentration: Trait Grazer/scavengers	-0.007	0.026	-0.264	0.792 [d)]
Concentration: Trait Facultative deposit feeders	-0.028	0.031	-0.902	0.367 [d)]
Concentration: Trait Filter feeders	-0.212	0.040	-5.298	**1.17E-07** *** [d)]

Significant findings are (p < 0.5) are highlighted in bold. Significant codes: <0.0001 ‘***’, < 0.001, ‘**’ < 0.01, ‘*’ < 0.05. The organisms *Lumbriculus variegatus* and *Chironomus riparius* were excluded from the analysis as they have different endpoints, reproduction and emergence, respectively.

[a)] Baseline.

[b)] The variable ‘concentration’ alone does not appear to be a significant predictor of mortality.

[c)] The variable ‘feeding trait’ alone does not appear to be a significant predictor of mortality.

[d)] The interaction term indicates whether one of the ‘feeding traits’ is more affected by the concentration of microplastics, which is statistically significant for the feeding trait Sediment/deposit feeder vs Filter feeders.

Table S13b. Estimated coefficients of the Generalized Linear Mixed Model with binomial distribution, with response variable Mortality, and explanatory variables ‘Concentration’ and “Feeding trait” with four levels; Filter feeders, Sediment/deposit feeder, Sediment grazer/scavengers, Facultative deposit feeders, see table S12. The group Grazer/scavengers is used as a reference to compare the other groups.

Coefficients:	Estimate	Std. Error	z value	Pr(>|z|)
Intercept	-0.898	0.495	-1.815	0.070 [a)]
Concentration	-0.002	0.019	-0.119	0.905 [b)]
Trait Sediment/deposit feeder	-0.774	0.627	-1.234	0.217 [c)]
Trait Facultative deposit feeders	-1.277	0.664	-1.923	0.055 [c)]
Trait Filter feeders	-0.255	0.703	-0.363	0.717 [c)]
Concentration: Trait Sediment/deposit feeder	0.007	0.026	0.264	0.792 [d)]
Concentration: Trait Facultative deposit feeders	-0.022	0.032	-0.666	0.506 [d)]
Concentration: Trait Filter feeders	-0.205	0.041	-5.031	**4.87E-07** *** [d)]

Significant findings are (p < 0.5) are highlighted in bold. Significant codes: <0.0001 ‘***’, < 0.001, ‘**’ < 0.01, ‘*’ < 0.05. The organisms *Lumbriculus variegatus* and *Chironomus riparius* were excluded from the analysis as they have different endpoints, reproduction and emergence, respectively.

[a)] Baseline.

[b)] The variable ‘concentration’ alone does not appear to be a significant predictor of mortality.

[c)] The variable ‘feeding trait’ alone does not appear to be a significant predictor of mortality.

[d)] The interaction term indicates whether one of the ‘feeding traits’ is more affected by the concentration of microplastics, which is statistically significant for the feeding trait Grazer/scavengers vs Filter feeders.

Table S13c. Estimated coefficients of the Generalized Linear Mixed Model with binomial distribution, with response variable Mortality, and explanatory variables 'Concentration' and "Feeding trait" with four levels; Filter feeders, Sediment/deposit feeder, Sediment grazer/scavengers, Facultative deposit feeders, see table S12. The group Filter feeders is used as a reference to compare the other groups.

Coefficients:	Estimate	Std. Error	z value	Pr(>|z|)
Intercept	-1.153	0.500	-2.307	0.021 * [a]
Concentration	-0.207	0.036	-5.750	**8.93E-09** *** [b]
Trait Sediment grazer/scavengers	0.255	0.703	0.363	0.716 [c]
Trait Sediment/deposit feeder	-0.518	0.631	-0.821	0.412 [c]
Trait Facultative deposit feeders	-1.022	0.668	-1.529	0.126 [c]
Concentration: Trait Sediment Grazer/scavengers	0.205	0.041	5.031	**4.87E-07** *** [d]
Concentration: Trait Sediment/deposit feeder	0.212	0.040	5.298	**1.17E-07** *** [d]
Concentration: Facultative deposit feeders	0.183	0.045	4.113	**3.90E-05** *** [d]

Significant findings are ($p < 0.5$) are highlighted in bold. Significant codes: <0.0001 '***', < 0.001, '**' < 0.01, '*' < 0.05. The organisms *Lumbriculus variegatus* and *Chironomus riparius* were excluded from the analysis as they have different endpoints, reproduction and emergence, respectively.

[a] Baseline.

[b] The variable 'concentration' alone does is a significant predictor of mortality for the group Filter feeders.

[c] The variable 'feeding trait' alone does not appear to be a significant predictor of mortality.

[d] The interaction term indicates whether one of the 'feeding traits' is more affected by the concentration of microplastics, which is statistically significant for the feeding trait Filter feeders vs Sediment grazer/scavengers, Sediment/deposit feeder and Facultative deposit feeders.

Table S13d. Estimated coefficients of the Generalized Linear Mixed Model with binomial distribution, with response variable Mortality, and explanatory variables 'Concentration' and "Feeding trait" with four levels; Filter feeders, Sediment/deposit feeder, Sediment grazer/scavengers, Facultative deposit feeders, see table S12. The group Facultative deposit feeders is used as a reference to compare the other groups.

Coefficients:	Estimate	Std. Error	z value	Pr(>|z|)
Intercept	-2.175	0.443	-4.905	9.33e-07 *** [a]
Concentration	-0.024	0.026	-0.907	0.364 [b]
Trait Filter feeders	1.022	0.668	1.529	0.126 [c]
Trait Sediment grazer/scavengers	1.277	0.664	1.923	0.055 [c]
Trait Sediment/deposit feeder	0.504	0.588	0.857	0.391 [c]
Concentration: Trait Filter feeders	-0.183	0.045	-4.114	**3.89e-05** *** [d]
Concentration: Trait Sediment grazer/scavengers	0.0216	0.032	0.666	0.506 [d]
Concentration: Trait Sediment/deposit feeder	0.0283	0.031	0.902	0.367 [d]

Significant findings are (p < 0.5) are highlighted in bold. Significant codes: <0.0001 '***', < 0.001, '**' < 0.01, '*' < 0.05. The organisms *Lumbriculus variegatus* and *Chironomus riparius* were excluded from the analysis as they have different endpoints, reproduction and emergence, respectively.

[a] Baseline.

[b] The variable 'concentration' alone does not appear to be a significant predictor of mortality.

[c] The variable 'feeding trait' alone does not appear to be a significant predictor of mortality.

[d] The interaction term indicates whether one of the 'feeding traits' is more affected by the concentration of microplastics, which is statistically significant for the feeding trait Facultative deposit feeders vs Filter feeders.

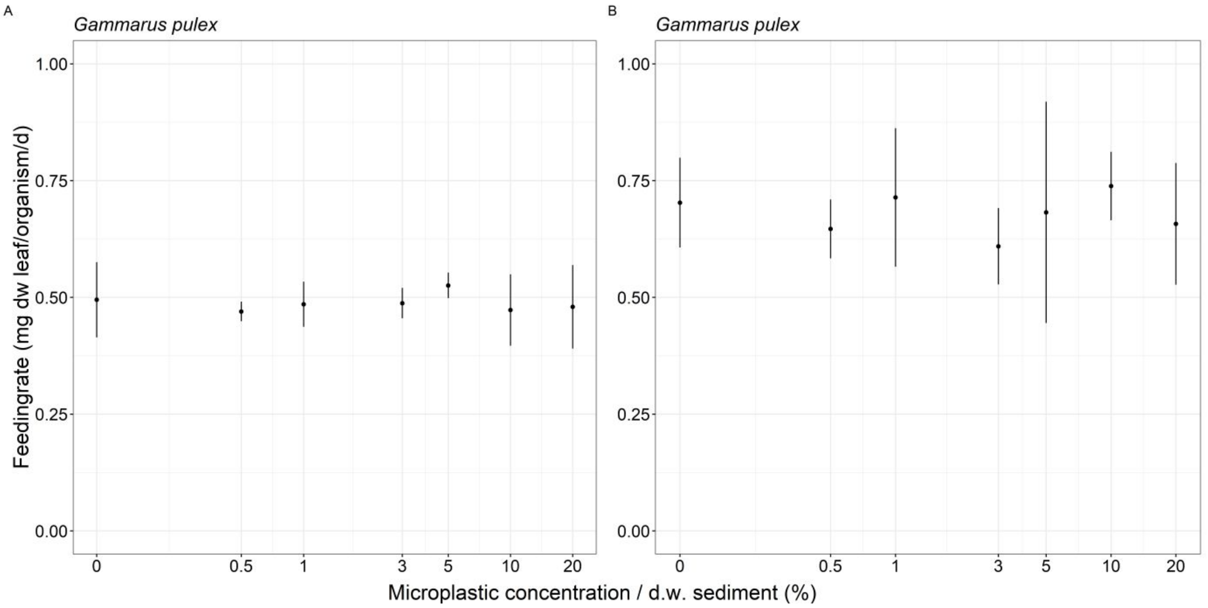

Figure S8. Mean feeding rate ± s.d. of *G. pulex*. expressed in mg dw leaf per organisms consumed during 28 days of exposure to A) PS and B) ERMP with concentrations up to 20% in sediment d.w.. Note that concentrations are on a log scale, additionally the zero concentration has been converted to 0.1 to allow plotting on the log scale.

References

(1) de Ruijter, V. N.; Redondo-Hasselerharm, P. E.; Gouin, T.; Koelmans, A. A. Quality Criteria for Microplastic Effect Studies in the Context of Risk Assessment: A Critical Review. *Environ. Sci. Technol.* **2020**, *54* (19), 11692-11705. DOI: 10.1021/acs.est.0c03057.

(2) Redondo-Hasselerharm, P. E.; Falahudin, D.; Peeters, E. T.; Koelmans, A. A. Microplastic Effect Thresholds for Freshwater Benthic Macroinvertebrates. *Environ. Sci. Technol.* **2018**, *52* (4), 2278-2286. DOI: 10.1021/acs.est.7b05367.

(3) Pan, C. G.; Mintenig, S. M.; Redondo-Hasselerharm, P. E.; Neijenhuis, P.; Yu, K. F.; Wang, Y. H.; Koelmans, A. A. Automated muFTIR Imaging Demonstrates Taxon-Specific and Selective Uptake of Microplastic by Freshwater Invertebrates. *Environmetnal Science & Technology* **2021**, *55* (14), 9916-9925. DOI: 10.1021/acs.est.1c03119.

(4) Primpke, S.; Lorenz, C.; Rascher-Friesenhausen, R.; Gerdts, G. An automated approach for microplastics analysis using focal plane array (FPA) FTIR microscopy and image analysis. *Anal. Methods* **2017**, *9* (9), 1499-1511. DOI: 10.1039/C6AY02476A

(5) Kooi, M.; Koelmans, A. A. Simplifying Microplastic via Continuous Probability Distributions for Size, Shape, and Density. *Environ. Sci. Technol. Lett.* **2019**, *6* (9), 551-557. DOI: 10.1021/acs.estlett.9b00379.

(6) Scircle, A.; Cizdziel, J. V.; Tisinger, L.; Anumol, T.; Robey, D. Occurrence of Microplastic Pollution at Oyster Reefs and Other Coastal Sites in the Mississippi Sound, USA: Impacts of Freshwater Inflows from Flooding. *Toxics* **2020**, *8* (2). DOI: 10.3390/toxics8020035.

(7) Koelmans, A. A.; Mohamed Nor, N. H.; Hermsen, E.; Kooi, M.; Mintenig, S. M.; De France, J. Microplastics in freshwaters and drinking water: Critical review and assessment of data quality. *Water Res.* **2019**, *155*, 410-422. DOI: 10.1016/j.watres.2019.02.054.

(8) Schneider, C. A.; Rasband, W. S.; Eliceiri, K. W. NIH Image to ImageJ: 25 years of image analysis. *Nat. Methods* **2012**, *9* (7), 671-675. DOI: 10.1038/nmeth.2089.

(9) Koelmans, A. A.; Besseling, E.; Foekema, E.; Kooi, M.; Mintenig, S.; Ossendorp, B. C.; Redondo-Hasselerharm, P. E.; Verschoor, A.; Van Wezel, A. P.; Scheffer, M. Risks of plastic debris: unravelling fact, opinion, perception, and belief. *Environ. Sci. Technol.* **2017**, *51* (20), 11513-11519. DOI: 10.1021/acs.est.7b02219.

(10) Koelmans, A. A.; Redondo-Hasselerharm, P. E.; Mohamed Nor, N. H.; Kooi, M. Solving the Nonalignment of Methods and Approaches Used in Microplastic Research to Consistently Characterize Risk. *Environ. Sci. Technol.* **2020**, *54* (19), 12307-12315. DOI: 10.1021/acs.est.0c02982.

(11) Kooi, M.; Primpke, S.; Mintenig, S. M.; Lorenz, C.; Gerdts, G.; Koelmans, A. A. Characterizing the multidimensionality of microplastics across environmental compartments. *Water Res.* **2021**, *202*, 117429. DOI: 10.1016/j.watres.2021.117429.

(12) Redondo-Hasselerharm, P. E.; de Ruijter, V. N.; Mintenig, S. M.; Verschoor, A.; Koelmans, A. A. Ingestion and Chronic Effects of Car Tire Tread Particles on Freshwater Benthic Macroinvertebrates. *Environ. Sci. Technol.* **2018**, *52* (23), 13986-13994. DOI: 10.1021/acs.est.8b05035.

(13) Ritz, C.; Baty, F.; Streibig, J. C.; Gerhard, D. Dose-Response Analysis Using R. *PLoS One* **2015**, *10* (12), e0146021. DOI: 10.1371/journal.pone.0146021.

(14) Zeileis, A.; Hothorn, T. Diagnostic Checking in Regression Relationships. *R News* **2002**, *2(3)*, 7--10.

(15) Bates, D.; Mächler, M.; Bolker, B.; Walker, S. Fitting Linear Mixed-E ects Models Using lme. *J. Stat. Softw.* **2015**, *67*, 1--48. DOI: 10.18637/jss.v067.i01.

(16) *RStudio: Integrated Development Environment for R*; Boston, MA, 2021. http://www.rstudio.com/ (accessed 02-11-2023).
